# Gallium-Based Liquid Metals: From Fundamental Properties to State-of-the-Art Applications

**DOI:** 10.3390/nano16030198

**Published:** 2026-02-02

**Authors:** Min Zhang, Peiying Liao, Yuanming Cao, Tingting Sun, Xuanyong Liu

**Affiliations:** 1 State Key Laboratory of Advanced Fiber Materials, College of Biological Science and Medical Engineering, Donghua University, Shanghai 201620, China; 2221031@mail.dhu.edu.cn (M.Z.); liaopeiying9@163.com (P.L.); 1229795@mail.dhu.edu.cn (Y.C.); 2 State Key Laboratory of High Performance Ceramics, Shanghai Institute of Ceramics, Chinese Academy of Sciences, Shanghai 200050, China

**Keywords:** gallium-based liquid metals, fundamental concepts, composite strategies, applications in flexible electronics

## Abstract

The rapid advancement of flexible and stretchable electronics has raised new demands for conductive materials with high conductivity and excellent mechanical properties. Compared to traditional conductive materials, gallium-based liquid metals exhibit a compelling set of attributes—including intrinsic deformability, high conductivity, good thermal conductivity, and a liquid state at or near room temperature—that address the critical requirements for conductors in flexible and stretchable electronics. However, the broader application of gallium-based liquid metals is limited by intrinsic challenges, such as oxidation tendency and high surface tension, while their multifunctional potential remains to be fully explored and developed. This paper provides a comprehensive review of gallium-based liquid metals, spanning from their fundamental concepts including intrinsic properties and processing characteristics (oxidative layer/droplet engineering) and functionalization techniques to their diverse applications in flexible electronics. It concisely summarizes key factors, existing issues, and challenges encountered during the design, research, and application of gallium-based liquid metals, aiming to provide guidance and assistance for subsequent research and applications.

## 1. Introduction

In the field of flexible electronics, ingenious structures such as wave structures, island-bridge structures, and 3D stretchable flexible structures have been designed to maintain stable electrical signals during bending or stretching. Gallium-based liquid metals demonstrate absolute advantages due to their inherent stretchability and high conductivity. With generally low melting points, they undergo phase transitions between liquid and solid states near or below room temperature. At room temperature, they exhibit low viscosity, fluidity, and deformability characteristic of liquids [1]. Simultaneously, its metallic nature creates numerous metallic bonds within droplets, resulting in high surface tension that promotes agglomeration and strong self-healing properties. Additionally, the ease of functionalization is a crucial characteristic of gallium-based liquid metal. It serves as a solvent for many metals and can be blended with diverse materials through various methods, broadening its application scenarios. Finally, a distinguishing feature of gallium-based liquid metals is their biocompatibility and low toxicity [2]. This further enables broader applications in fields demanding stringent biosafety standards, such as brain–computer interfaces and integrated diagnosis and treatment systems.

While gallium-based liquid metals possess inherent advantages, the self-limiting oxide film that spontaneously forms on their surface warrants particular attention in liquid metal research [3]. The oxidation levels can be increased by dispersing large volumes of liquid metal into smaller droplets, enhancing patterning capabilities but simultaneously significantly raising electrical resistance. Therefore, regulating this surface oxide layer to balance conductivity and patterning performance is crucial. During the patterning process via printing, liquid metals are micro-/nano-engineered to enhance adhesion and printing onto various substrates. When subsequently used as flexible circuits or electronic components, their conductivity becomes paramount. Thus, printed liquid metals require conductive activation—i.e., sintering—to achieve both properties.

Additionally, gallium-based liquid metals can be blended with diverse materials to create multifunctional composites. The blending strategies based on the properties of added materials can be categorized into the following three broad approaches: physical adsorption, chemical bonding, and biomodification. Physical adsorption primarily involves inorganic molecules—such as carbon materials and inorganic oxides—and polymeric macromolecules. Chemical bonding mainly anchors liquid metals through functional groups of metallic elements or small organic molecules. As for biological modification, it focuses on binding biomacromolecules or biomolecules to liquid metals, further enhancing their biocompatibility and enabling functional applications within living organisms.

Finally, from a macro perspective, liquid metals hold extensive applications and development potential in the field of flexible electronics. For instance, their stretchable properties are demonstrated in printable flexible circuits, maintaining stable signal transmission during stretching [4]. In energy and battery technology, liquid metals can serve as electrodes and facilitate various self-powered energy generation methods while preventing dendrite growth in lithium and zinc batteries during cycling, thereby extending battery lifespan [5,6]. In wearable sensors, liquid metals’ conformability and ease of functionalization enhance sensor performance [7]. Within micro-robotics, their functionalizability enables diverse actuation modes [8]. Additionally, liquid metals demonstrate exceptional thermal management capabilities [9].

**Scheme 1:**
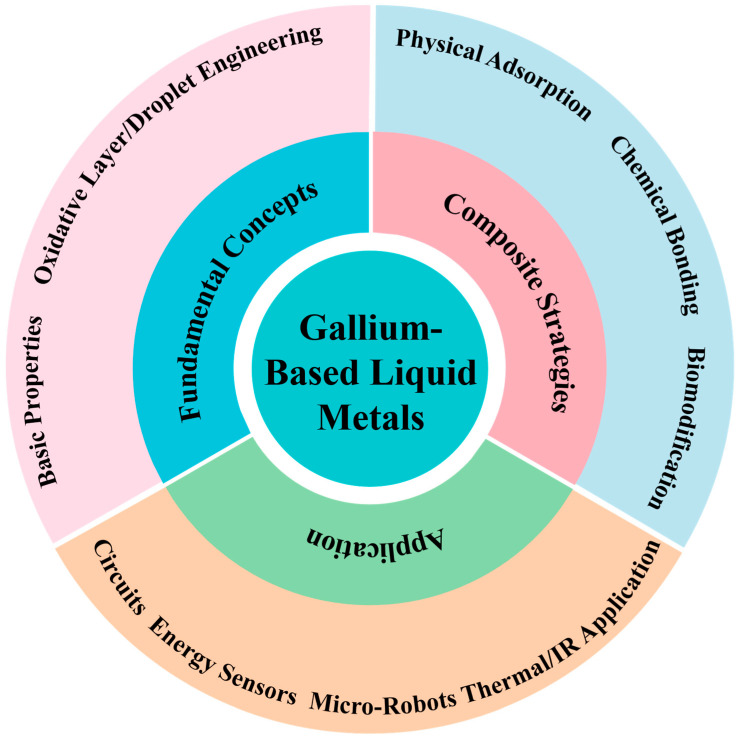
Overview of this review.

Numerous reviews have been conducted on liquid metals, covering their intrinsic properties, design and fabrication strategies for liquid metal composites, and applications across diverse fields. This review is structured around a logical framework (Scheme 1) from the intrinsic properties of liquid metals, through processing characteristics (oxidative layer/droplet engineering), to functional compounding, and finally to targeted applications involving printable flexible circuits, reconfigurable electronics, energy devices, sensors, soft robotics, and thermal/infrared management. The aim is to provide a more concise and clear design research approach for subsequent liquid metal studies.

## 2. Fundamental Concepts of Gallium-Based Liquid Metals

Liquid metals, as the name suggests, are metals exhibiting fluid properties. To some extent, any metal in its molten state qualifies as a liquid metal. However, metals generally possess high melting points—common metals like iron (1538 °C) and copper (1085 °C) both exceed 1000 °C. Reaching these melting points under ordinary conditions is challenging, severely limiting their modification and application in molten form. Therefore, broadly defined liquid metals are typically metallic materials with melting points below 300 °C, also known as low-melting-point metals. Excluding alkali metals with special properties, the elements composing liquid metals are primarily late transition metals (Ga, In, Tl, Sn, Pb, Al, and Bi) and zinc group elements (Zn, Cd, and Hg) [10]. Mercury, the most familiar liquid metal with a melting point of −38.8 °C, readily vaporizes into mercury vapor and exhibits significant toxicity. In contrast, gallium (Ga, 29.8 °C), indium (In, 156.6 °C), tin (Sn, 232 °C), and bismuth (Bi, 271.4 °C) meet the requirement of melting below 300 °C and are commonly considered for liquid metal composite materials.

In the narrow sense, liquid metals refer to metals or alloys that remain liquid under standard temperature and pressure conditions. Gallium’s uniqueness lies in its ability to reduce Gibbs free energy through negative mixing enthalpy with other metals [11,12], enabling it to integrate multiple metals at lower temperatures and serving as an excellent “metallic solvent.” Furthermore, gallium possesses a lower melting point than other post-transition metals, and certain gallium-based alloys can achieve a liquid state at room temperature. Their advantageous characteristics include, but are not limited to, metallic properties [13] (high electrical and thermal conductivity), phase transitions near room temperature [14,15], low viscosity [16] (flowability [17] and deformability [1]), high surface tension (self-healing capability [18,19]), catalytic activity [20,21] (self-limiting surface oxidation), ease of functionalization [22,23], stimulus responsiveness [24,25] (capable of loading and mixing with diverse materials), biocompatibility [2], and low toxicity [26] (relative in vivo safety). The following sections provide a detailed discussion from the following two perspectives: the basic properties and characteristics of liquid metals and their processing characteristics, which include oxidative layer and micro-/nanodroplets engineering.

### 2.1. Basic Properties and Characteristics

#### 2.1.1. Low Melting Point

The most intuitive property of liquid metals and their alloys is their low melting point. While possessing a low melting point, they also exhibit a high boiling point, resulting in a wide liquid temperature range. As shown in Table 1, by adjusting the ratio of metal content, properties such as melting point, density, surface tension, electrical conductivity, thermal conductivity, etc., can be controlled [27,28,29,30], enabling the selection of more suitable liquid metals and their alloys for various application fields. As can be seen, Bi and bismuth-based metals [31] possess higher melting points than Ga and gallium-based liquid metals, making Bi_35_In_48.6_Sn_15.9_Zn_0.4_ alloys suitable for high-temperature extrusion-based 3D printing process. When fabricated devices do not require room-temperature flowability but necessitate lower processing temperatures and functional phase transitions at reduced temperatures, bismuth-based metals should be considered [32]. In contrast, gallium liquid metals have a lower melting point than other post-transition metals, and when alloyed with other metals, their melting point can be decreased further, such as gallium–indium alloys and gallium–indium–tin alloys. Different metal alloys are named and distinguished based on elemental composition. Currently, the most researched and utilized gallium-based liquid metals are Ga_75_In_25_ (EGaIn) and Ga_68.5_In_21.5_Sn_10_ (Galinstan) [33]. This low melting point of gallium-based liquid metals renders them highly suitable for room-temperature flexible and wearable electronics.

**Table 1:** Table summarizing basic properties and characteristics of various typical liquid metals [31,34,35,36,37,38,39,40].

Alloyed Elements	Melting Point (°C)	Boiling Point (°C)	Density (g·cm^−3^)	Electrical Conductivity (10^6^ S·m^−1^)	Thermal Conductivity (W·m^−1^·K^−1^)	Surface Tension (mN·m^−1^)	Viscosity (10^3^ Pa·s)
Ga	29.8	2204.8	5.91	7.4	40.6	711 (30 °C)	1.37
In	156.6	2023.8	7.31	12.5	81.6	556 (157 °C)	1.75
Sn	231.9	2622.8	7.29	8.7	66.6	561.6 (232 °C)	1.86
Bi	271.4	1556.8	9.79	0.9	7.87	270 (382 °C)	/
Ga_75.5_In_24.5_ (EGaIn)	15.4	2000	6.28	3.4	26.4	624 (22 °C)	1.99
Ga_68.5_In_21.5_Sn_10_ (Galinstan)	13.2	/	6.4	3.0	23.1	/	2.4
Bi_32.5_In_51_Sn_16.5_	62.0	/	7.55 (99 °C)	/	14.5	417 (85 °C)	/
Bi_35_In_48.6_Sn_15.9_Zn_0.4_	58.3	/	7.90	/	23.05	/	/

#### 2.1.2. High Electrical Conductivity and High Thermal Conductivity

Compared to many flexible conductive polymers and conductive nanofillers, LM exhibits superior electrical conductivity, which is crucial for electrical applications. However, due to functionalization requirements and material processing methods, conductivity may deteriorate. Adding small amounts of precious metal nanoparticles can enhance conductivity while also serving as a means of conductive activation [41].

Alongside its high conductivity, LM’s superior thermal conductivity makes it widely applicable in thermal management. Liquid metal is engineered for chip thermal management, battery thermal management, and other applications, which will be detailed in subsequent application sections.

#### 2.1.3. Low-Viscosity Liquid Flowability and Deformability

The viscosity of liquid metals is closely related to temperature. The oxide layer confers non-Newtonian rheological properties to liquid metals [42], thereby facilitating their processing and patterning [43], and plays a dominant role in their rheological behavior. Hydroxyl groups on the oxide layer surface can exert forces on different substrates and ligands, altering the wettability of liquid metals to some extent [44]. Under high humidity conditions, the oxide layer thickness of gallium-based liquid metals increases, leading to higher viscosity and enhanced non-Newtonian rheological response. The regulation of the oxide layer is detailed in the section on processing characteristics.

Beyond modifying rheological properties through surface oxide layer control, substrate treatment can also regulate surface adhesion. For instance, texturing can create metal-repellent surfaces [45,46]. Alternatively, metals like gold [47], silver, or copper can serve as alloy layers, allowing liquid metal to wet surfaces via coalescence and enabling the fabrication of stretchable biphasic solid–liquid thin metal films.

Embedding nanoparticles into metal droplets can also modify the rheological properties of liquid metals. For instance, adding copper fillers can regulate the rheological properties of the liquid metal composite material (Cu-EGaIn), and at the same time, it can also improve the thermal conductivity, electrical conductivity and wettability [48,49,50]. Incorporating hollow glass spheres enables the preparation of liquid metal putty. Mixing polymers with liquid metals yields composite stretchable conductors.

#### 2.1.4. High Surface Tension and Self-Healing Properties

Due to its inherent metallic properties, liquid metal droplets contain a large number of metallic bonds, resulting in relatively high surface tension. Consequently, under normal temperature and pressure conditions without special treatment, liquid metal droplets typically coalesce into spherical droplets to minimize surface energy. Concurrently, this property also endows liquid metals with self-healing capabilities [51,52]. When dispersed into smaller droplets, external influences can still restore them into a larger, cohesive mass. Typically, strain can cause the oxide layer to rupture.

#### 2.1.5. Low Toxicity and Biocompatibility

Liquid metals demonstrate excellent biocompatibility and low toxicity in experiments when functionalized with minimal doses of non-toxic elements, avoiding the use of highly reactive or toxic substances like Hg or Pb. They are not only employed in surface materials such as biosensors and brain–computer interfaces but also engineered for drug delivery and therapeutic applications.

#### 2.1.6. Safety and Compatibility

Beyond hybridization among late transition metals, gallium can also combine with other transition metals, enhancing the multifunctionality of liquid metals and broadening their fundamental property range. Due to gallium’s strong reducing power, it reacts with copper (Ga + Cu → CuGa_2_) to form intermetallic compounds [53]. Also, without any external stress, liquid metal can also damage the passivation layer on the surface of aluminum particles, thereby eroding the grain boundaries of the alumi-num core, transforming the aluminum-aluminum interface into an aluminum-gallium interface, and resulting in a decrease in the binding energy. This phenomenon is known as liquid metal embrittlement [54]. The aforementioned LM “corrosion” of other metals can reduce their mechanical strength. Therefore, preventing liquid metal leakage is crucial to avoid corrosion and damage to other electronic device components. Typically, encapsulating liquid metal with polymeric materials ensures flexibility and stretchability while preventing leakage and maintaining the integrity of conductive circuits.

### 2.2. Processing Characteristics

#### 2.2.1. Surface Oxide Layers Formation and Regulation

**Figure 1:**
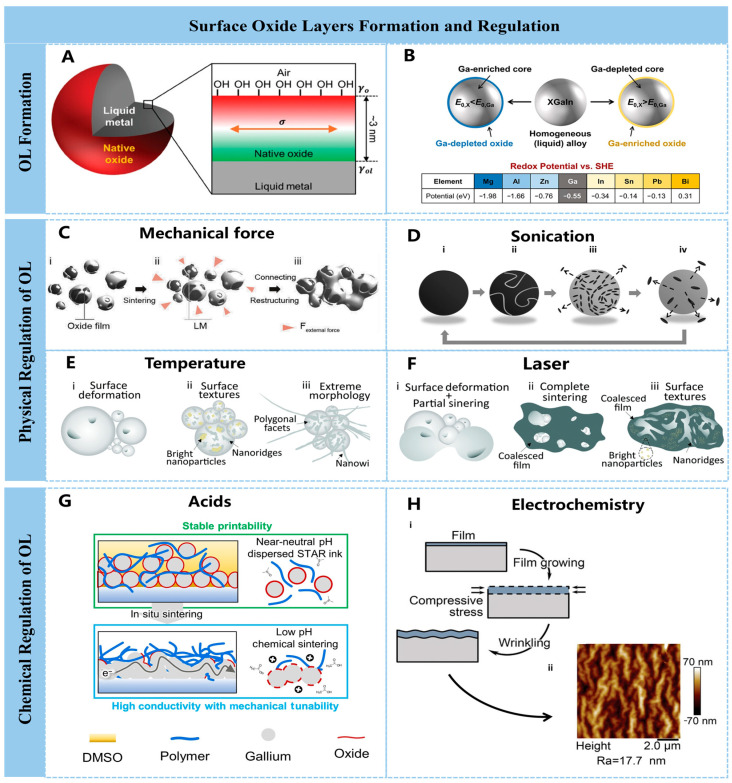
Surface oxide layers formation and regulation. (**A**) Schematic of the native oxide layer on liquid metal [56]. Copyright 2024, John Wiley and Sons. (**B**) Oneline summary−redox potential dictates bidirectional Ga segregation between shell and core in a homogeneous Ga−based alloy [61]. Copyright 2023, American Chemical Society. (**C**) Mechanical strain ruptures the brittle Ga_2_O_3_ shell, extruding liquid metal cores to establish continuous conductive pathways [65]. Copyright 2025, John Wiley and Sons. (**D**) Schematic images of LM/MO spherical structures during sonication. Gray shapes, black shapes, and black ovals stand for galinstan spheres, oxide layers, and nanoplatelets, respectively, (i) The stage where a thin oxide layer forms on the surface; (ii) Oxide layer deformation and wrinkling stage; (iii) The stage where the oxide layer breaks and detaches from the surface; (iv) Under a longer ultrasonic treatment time, the oxidized nanosheets detach and become thicker [66]. Copyright 2014, John Wiley and Sons. (**E**) Surface morphologies of liquid metal nanoparticle films after thermal sintering [69]. Copyright 2019, RSC Pub. (**F**) Surface morphologies of liquid metal nanoparticle films after laser sintering [69]. Copyright 2019, RSC Pub. (**G**) Schematic illustration of the sintering process of STAR ink with activated DMSO. The acidic activated DMSO reduces the oxide on dispersed gallium microparticles and induces their sintering [75]. Copyright © 2025, The American Association for the Advancement of Science. (**H**) (i) Schematic diagram of electrochemically induced liquid metal surface wrinkles by growth stress; (ii) The atomic force microscope (AFM) image of the planar surface of the wrinkled sample [76]. Copyright 2023, John Wiley and Sons.

Oxygen concentrations as low as a few ppm are sufficient to induce the formation of oxide layers (OLs) on liquid metal surfaces, with thicknesses reaching the atomic scale [55], whose growth is limited to ~3 nm at room temperature [56,57] (Figure 1A). Particularly, the surface of gallium-based liquid metals generally spontaneously forms an amorphous Ga_2_O_3_ oxide film. This occurs because its Gibbs free energy of formation is lower than that of most metal oxides [58]. The growth process can be described by the Cabrera–Mott oxidation model at low to moderate temperatures [59], where an initial oxide monolayer (Langmuir layer) forms via rapid reaction, followed by slow growth driven by the reduction in adsorbed oxygen at the oxide interface [60]. Surface oxides typically form from the oxide with the lowest Gibbs free energy. Thus, when liquid metals contain alloying elements with redox potentials higher than gallium’s, the oxide surface (shell) is expected to be gallium-rich. Conversely, surfaces are expected to be enriched with oxides derived from the alloying elements [61] (Figure 1B). The oxide layer referred to in the remainder of this paper is primarily gallium oxide.

Notably, the oxide layer has a dual impact. On the one hand, the thin oxide layer protects the metal from further oxidation and provides anchoring sites for surface functionalization [62]. Also, this layer reduces the surface tension of liquid metal, enhancing its adhesion to substrates, which will facilitate the patterning of liquid metal and simplify the fabrication of related electronic devices [63]. However, on the other hand, the oxide layer has a detrimental effect on electrical properties. Despite the negligible impact of the native oxide layer due to its ultrathinness, the accumulation of particle oxide layers in a film formed by LM particle creates critical electrical barriers that cause severe performance degradation, an effect that cannot be ignored. Given this scenario, removal of the oxide layer is necessary. Thereby, the on-demand regulation of surface oxides is crucial [64].

Accordingly, the on-demand regulation of surface oxides can be broadly categorized into the following two parts: on the one hand, enhancing oxide layer numbers during flexible material fabrication to ensure optimal adhesion and high-precision patterning or device formation; on the other hand, appropriately removing the surface oxide layers to conductively activate the liquid metal droplets and to establish electrical pathways, preventing the insulating nature of these oxide layers from hindering device functionality. The strategies to control the oxides layers typically involve chemical regulation and physical regulation, which are introduced in detail below.

Primary methods for regulation of oxides layer include pressing or scraping, localized ultrasonication, laser sintering, and temperature control. As shown in Figure 1C, the external force can induce microcracks in the brittle oxide layer and thereby facilitate the metal-to-metal by releasing the LM core [65]. As displayed in Figure 1D, during sonication, the morphological transformation, as well as the oxide layers of the LM, undergoes four stages [66]. Under prolonged sonication from stage (i) to stage (ii), the oxide layer undergoes a transition from a thin native layer to an increasingly thickened one, combined with wrinkles and eventually cracks. Further prolonging the sonication time, the oxide layer is detached from the surface (stage iii) and forms small planar residue. Then, this residue will become thicker and is simultaneously detached, forming a new oxide layer. The mechanical force method is a simple strategy to regulate the OL, and the forces (pressure) acting on the LM surface or forces (tension) whin plane have both been effective for the regulation of OL [67,68]. Temperature and laser irradiation serve as effective means for regulating the oxide layer [69]. As shown in Figure 1(Ei,Fi), when laser and thermal energy are applied, the oxide layer thickens due to the increased energy. With the increased thermal laser energy, owing to the different thermal expansion of the LM and OL, the oxide layer shows extensive cracking, as shown in Figure 1(Eii,Fii). When the temperature is further increased, fiber-shaped particle morphology is observed, as shown in Figure 1(Eiii). On the other hand, when the laser irradiation energy is further increased, owing to phase segregation and localized oxide fracture, two types of structures of large nanocrystals and small, bright nanoparticles are observed on the laser-coalesced film, as seen in Figure 1(Fiii). The difference mainly originates from the fact that high temperature induces lower thermal stress, yet it causes more oxidation than local laser irradiation. As a result, compared to thermal processing, laser irradiation is more effective in suppressing oxidation and achieving superior electrical conductivity [69,70]

The aforementioned methods such as ultrasonication, laser irradiation, and thermal treatment may adversely affect the flexible substrates and, in particular, cause damage to printed LM patterns. Thereby, these methods are not well-suited for flexible printing processes. In contrast, the chemical regulation of OL, including acidic/alkaline treatment and electrochemical control, demonstrates promising application prospects.

Acid (i.e., hydrochloric acid (HCl) and acetic acid) treatment has been proven effective in removing OL [71,72,73]. The simplest direct method involves treating the surface with hydrochloric acid vapor to remove the oxide layer. Ruizhe Xing et al. studied the influence of pH on the EGaIn and thereby the metallic wetting properties between EGaIn and Cu particles. It was found that the OL can be stable in the condition of pH between ~3 and 11, and the OL can be removed when the pH of the ink composed of water, liquid metal, and copper is adjusted to 1, promoting wettability between the LM and Cu and thereby endowing the ink with the necessary fluidity for printing. The electrical conductivity of the printed material after drying can reach 1.05 × 10^5^ S m^−1^ without any additional treatment [74]. Simok Lee et al. ingeniously utilized the acidic products (such as formic acid, acetic acid, methanesulfonic acid and sulfuric acid) generated by the decomposition of dimethyl sulfoxide (DMSO) during the ultrasonic treatment at the tip of the ink droplet, achieving in-situ acid removal of the oxide layer (OL) in the ink (Figure 1G). Owing to this pH control method, the final printing transformative electronic systems displayed exceptional mechanical tunability (tuning ratio 1465) and electrical conductivity (2.27 × 10^6^ S m^−1^) [75]. Electrochemistry represents another effective method for modulating oxide layers. Studies have demonstrated that electrochemical approaches can both remove and thicken the OL. Zhuquan Zhou et al. use LM as the anode reactant in an electrolyte solution of carboxymethylcellulose sodium, thereby regulating the thickness of OL from 1 to 5 nm (formed spontaneously in air) to hundreds of nanometers via electrochemical oxidation [76]. As further shown in Figure 1(Hi), the volume expansion occurred in the OL during anodization, and the OL buckled to release the stress seen in Figure 1(Hii), forming micro-wrinkles when LM was re-oxidized. It was also found that the wavelength of these micro-wrinkles increased with voltage, while they first increased and then decreased with increasing electrolyte concentration.

#### 2.2.2. Liquid Metal Micro-/Nanodroplets Engineering

To enhance the adhesion of liquid metal and thereby facilitate its functionalization [77], bulk LM requires micro-/nanoscale processing to transform it into low-dimensional liquid metal structures. Currently, multiple techniques have been successfully employed to generate liquid metal micro-/nanodroplets, including mixing under shear agitation [78], ultrasonic irradiation, microfluidic droplet formation, and splashing jets.

**Figure 2:**
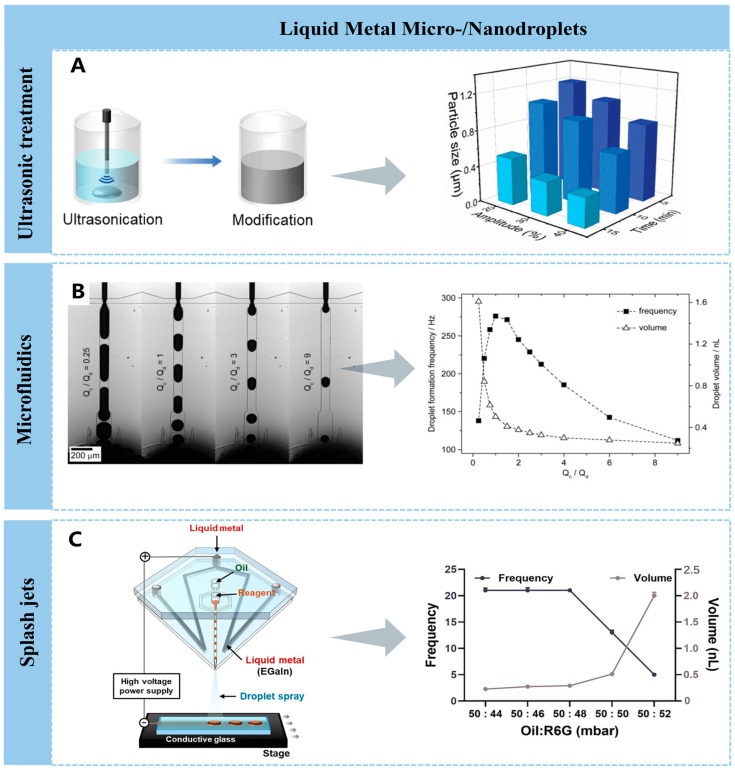
LM micro-/nanodroplets engineering. (**A**) Schematic illustration of the preparation of modified LMP stock solution and the average LMP diameters obtained under varying ultrasonication amplitudes (20–40%) and durations (5–15 min) [80]. Copyright 2024, John Wiley and Sons. (**B**) Liquid metal droplet formation in aqueous PEG solution using a device with 40 μm nozzle geometry. Formation frequency and volume of liquid metal droplets vs. flow-rate ratio Q_c_/Q_d_ [86]. Copyright 2012, John Wiley and Sons. (**C**) Liquid metal-embedded electrospray deposition (LM-ESD) system [89], the chart pointed to by the arrow shows the quantitative analysis of the frequency of droplet generation under different flow conditions (on the left axis) and the estimated volume of the droplets (on the right axis). Copyright 2025, John Wiley and Sons.

Ultrasonic treatment is a common method for breaking down bulk liquid metal into small particles. The cavitation effect generates high-intensity shear forces that overcome the high surface tension of liquid metal, causing it to fracture into micro-/nanoscale droplets [79]. During sonication in air, gallium oxide continuously forms, fractures, and reforms, leading to a decrease in the size of LM particles as sonication time and amplitude increase (Figure 2A) [80]. The ultrasonic treatment results in the formation of LM nanoparticles with a wide distribution of geometric dimensions. Studies have shown that surfactants such as polyvinylpyrrolidone (PVP), thiol, etc., can stabilize and prevent coalescence of LM particles and achieve size uniformity in these particles [81,82]. Extending processing time typically reduces droplet size [83], but it also broadens the size distribution. Excessive processing may cause over-oxidation of the liquid metal, generating additional rod-like oxides GaO(OH). In addition, the ultrasonic parameters such as power, amplitude, temperature, and solvent directly influence droplet size and uniformity [81,84]. Therefore, selecting appropriate parameters and processing duration is essential.

Microfluidics, a low-cost and easily fabricated method, enables rapid production of liquid metal nanoparticles with tunable size distributions [85]. Tanya Hutter et al. reported a microfluidic channel that could generate and control the LM microdroplets via changing the flow-rate ratio of continuous phase (Q_c_) and dispersed droplet phase (Q_d_), as shown in Figure 2B. The droplet volumes decreased from 1.6 nL to 0.25 nL when the flow-rate ratios increased from Q_c_/Q_d_ = 0.25 to Q_c_/Q_d_ = 9 [86]. In addition, the microfluidics strategy can also be combined with electrophoresis to form an uneven electric field around LM droplets and induce charges on their surfaces, thereby driving droplets within the electric field—a flexible approach for LM droplet preparation [87].

In addition, splash jets overcome the high surface tension of liquid metals through shear forces generated by high-velocity jets and interfacial instabilities (e.g., Plateau–Rayleigh instability), fragmenting them into micro- and nano-sized droplets. Electro- or laser-induced jet dynamic fracture [88], supplemented by oxide layer regulation, enables droplet size uniformity (10 nm–100 μm). Chaeyeong Kang et al. presented a liquid metal-embedded electrospray deposition (LM-ESD) system, as shown in Figure 2C, where the reagent and oil phases in separate inlets could generate microscale water-in-oil droplets via hydrodynamic shearing at the junction. Also, the droplet size and generation frequency could be controlled by the flow rates of reagent and oil, as well as the nozzle-to-substrate distance, nozzle diameter, and applied voltage [89].

Although the strategies and the natural oxide layer contribute to the formation of LM micro-/nanodroplets, the particle size further increases due to coalescence and Ostwald ripening, making it difficult to maintain the stability of the micro-/nanodroplets [90]. Further regulating the surface oxide layer and utilizing methods such as ligand modification and polymer grafting could control its stability.

## 3. Composite

An overview of the fundamental properties of liquid metals reveals that despite their high electrical conductivity and deformability, they face challenges in standalone applications. This section further outlines strategies for achieving multifunctionality through composite liquid metals, including physical adsorption, chemical bonding, and biomodification.

### 3.1. Physical Adsorption

Many non-metallic substances can undergo electrostatic adsorption onto the oxide layer of liquid metal surfaces, allowing them to physically adsorb into mixtures through simple stirring or uniform dispersion in solvents.

**Figure 3:**
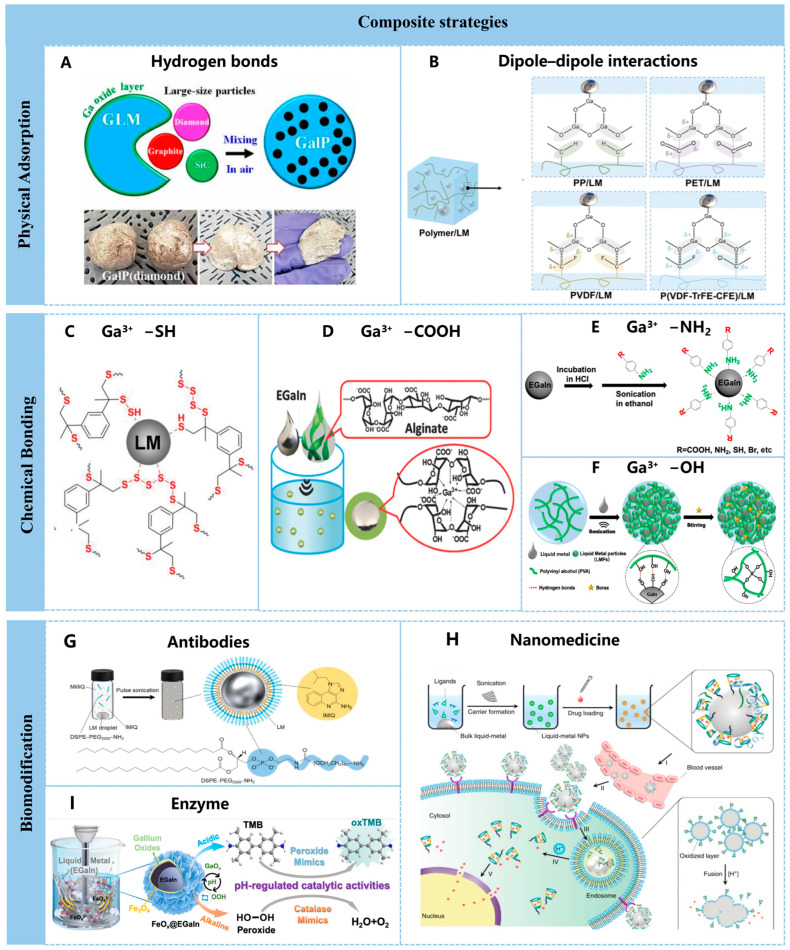
Functional material loading of liquid metal. (**A**) Schematic illustration of showing the fabrication of composite materials composites by mixing graphite flakes, diamond, or SiC particles with gallium-based liquid metal (GLM) [91]. Copyright © 2021, The American Association for the Advancement of Science. (**B**) Schematic diagram of interfacial interactions of PP/LM, PET/LM, PVDF/LM, and P(VDF-TrFE-CFE)/LM composites [93]. Copyright © 2023, Royal Society Of Chemistry. (**C**) Scheme of the surface interactions between LM droplets with polysulfide loops (R-Sn-R) and thiol terminal groups (R-SH) [94]. Copyright 2019, John Wiley and Sons. (**D**) Schematic illustration of sonicating EGaIn in alginate solution, with EGaIn droplets encapsulated by alginate microgel shells [95]. Copyright 2018, John Wiley and Sons. (**E**) Schematic process for the preparation of EGaIn modified with p-aniline derivatives [96]. Copyright 2022,American Chemical Society. (**F**) The hydroxyl groups of PVA stabilize LMP through hydrogen bonding interactions [99]. Copyright 2019, American Chemical Society. (**G**) Schematic illustration of the preparation of PEG–IMIQ–LM nanoparticles [101]. Copyright 2023, John Wiley and Sons. (**H**) Schematic design of liquid metal nanoparticles loaded with doxorubicin for targeted cancer therapy [102]. Copyright 2015, Springer Nature. (**I**) Schematic illustration of sonicating EGaIn in alginate solution, with EGaIn droplets encapsulated by alginate microgel shells [103]. Copyright 2025, John Wiley and Sons.

Common examples include physical wetting with carbon materials such as graphene, diamond, and silicon carbide (Figure 3A) [91], where the oxygen-containing groups of graphene oxide (GO) could coordinate with Ga^3+^ ions to form hydrogen bonds. Inorganic oxide carriers (SiO_2_) adsorb onto LM via van der Waals forces or hydrogen bonds without forming strong chemical bonds. Bonding strength can be enhanced through bridging with surface silane coupling agents (e.g., APTES) [92], forming a liquid metal-silica gel ink (LMSG) with high conductivity, excellent thermal stability, high flexibility, and good surface compatibility. LMSG ink also exhibits a conductive flow threshold as low as 43 wt%, requires no sintering process to form conductive pathways, and demonstrates broad surface compatibility with materials like plastics, wood, and skin. Highly flexible and complex integrated circuits incorporating microcontroller units can be successfully fabricated.

In addition, interfacial interactions are a key issue limiting improvements in electrical properties and energy density. Yuhang Li et al. demonstrated that increased polymer polarity significantly enhances dipole–dipole interactions between polymer polar groups and the oxide layer of LM. The following polymers exhibit an order of polarity: polypropylene (PP)/LM < polyethylene terephthalate (PET)/LM < polyvinylidene fluoride (PVDF)/LM < poly(vinylidene fluoride-trifluoroethylene-chlorofluoroethylene) (P(VDF-TrFE-CFE) /LM (Figure 3B) [93], in which the most polar PVDF/LM composite exhibits favorable interfacial interactions that suppress dielectric losses, enabling higher capacitive energy storage density (+44%, 1.68 J cm^−2^) without compromising energy efficiency (80%).

### 3.2. Chemical Bonding

In addition to the formation of eutectics or solid solutions through melting between metals as mentioned above, liquid gallium can also form chemical bonds with numerous materials via surface-bound gallium oxide, such as hydroxyl, carboxyl, thiol, and amino groups. The oxide layer on gallium–indium alloy surfaces can be chemically modified to graft organic compounds.

Ga^3+^ ions in the thiol (−SH) oxide layer form Ga−S bonds with thiols (Figure 3C) [94], significantly enhancing the dispersion and interfacial adhesion of liquid metal within composite materials to achieve self-assembled monolayers. Grafting thiol surfactants (e.g., dodecyl mercaptan) onto liquid metal surfaces not only suppresses oxidation but also promotes stable binding of magnetic particles to the liquid metal via polydopamine coatings, forming functional magnetorheological fluids.

The carboxyl (−COOH) groups form coordination bonds with gallium oxide. When EGaIn is sonicated in an alginate solution, the Ga^3+^ generated by oxidation of Ga interacts with the carboxyl groups in the alginate G−blocks, thereby producing an ionically cross-linked alginate hydrogel. During sonication, alginate adheres to EGaIn droplets, reduces their size, and simultaneously builds a robust hydrogel shell that prevents droplet coalescence (Figure 3D) [95].

The amino (−NH_2_) group also has a strong binding affinity for liquid metals. By modifying the surface of the eutectic gallium-indium (EGaIn) alloy with p-aminobenzoic acid derivatives, an oxide layer can be avoided from forming on the gallium-based liquid metal surface (Figure 3E) [96], thereby stabilizing EGaIn nanoparticles and significantly improving their conductivity. Additionally, different p-aminobenzoic acid derivatives can be used to introduce monolayer organic molecules with multiple functional groups [97], increasing their functionality and application diversity. Even long polyaniline organic molecular chains can be directly synthesized at the dynamic interface of the p-aminobenzoic acid-modified liquid metal [98], providing a new idea for the synthesis of liquid metal-polymer hybrid nanocomposite materials.

The hydroxyl groups (−OH) stabilize liquid metal particles through hydrogen bonding interactions, allowing the dynamic cross-links between the hydrogel and LMP to break and reversibly coalesce within the hydrogel network, thereby endowing the hydrogel with electrical and mechanical self-healing capabilities (Figure 3F) [99].

These chemical bonds can be extended to diverse materials, including organic substances, functional polymers, and hybrid materials. Additionally, polymer grafting enables the introduction of active groups (e.g., −OH, −COOH) onto the oxidized surface via plasma treatment, facilitating further bonding of polymer chains (e.g., polyethylene glycol PEG).

### 3.3. Biomodification

Liquid metals demonstrate broad application prospects in the biomedical field due to their low toxicity and excellent biocompatibility. By performing biomodification on liquid metal nanoparticles [100], their targeting capability, stability, stimulus responsiveness, and therapeutic efficacy can be significantly enhanced, providing innovative solutions for tumor diagnosis and treatment, drug delivery, and biosensing.

Modification with biological components enhances targeting efficacy in biological applications, enabling more precise action at specific sites. For instance, immobilizing monoclonal antibodies (such as anti-EGFR and anti-HER2) onto gallium–indium alloy surfaces via amide bond coupling or physical adsorption significantly boosts active targeting capabilities toward tumor cells. Antigen-antibody specific binding drives the accumulation of liquid metal nanoparticles at tumor sites, enhancing drug delivery efficiency. A team from Japan’s Japan Advanced Institute of Science and Technology (JAIST) co-adsorbed PD-L1 antibodies-which block immunosuppressive signals—onto gallium–indium alloy nanoparticles, activating dendritic cell-derived imiquimod and the near-infrared fluorescent probe indocyanine green (Figure 3G) [101]. This design endows the nanoparticles with the following triple functions: tumor fluorescence imaging, near-infrared photothermal therapy, and immune activation.

Yue Lu et al. reported a transformable liquid metal nanomedicine based on a core–shell nanosphere-composed of a liquid-phase eutectic gallium–indium core and a thiolated polymeric shell-that can be simply produced through a sonication-mediated method. Also, this core–shell nanosphere liquid metal can be further loaded with doxorubicin (Dox) (Figure 3H) [102], thereby establishing a novel liquid metal-based drug delivery platform for anticancer therapy.

Yuhe Shen et al. harnessed pH as a tunable lever to direct the galvanic replacement potential, transforming iron-based liquid metal nanozymes (FeOx@EGaIn) into nanoflowers that couple inherent magnetism with switchable dual-enzyme catalysis (Figure 3I) [103]. The iron oxides exhibit different oxidation forms with changes in pH, resulting in the FeOx@EGaIn nanozymes showing excellent peroxidase (POD) catalytic activity under acidic conditions, while switching to catalase (CAT) activity in neutral to alkaline conditions and possessing high catalytic stability. This approach expands the application prospects of liquid metals in catalysis, sensing detection, and biomedical fields.

## 4. Applications in Flexible Electronics

Liquid metals, as metals with flow properties, hold an irreplaceable position in flexible electronics. Their applications in this field are primarily manifested in printable flexible circuits, energy and battery technologies, and sensor technologies. Liquid metals and their composites significantly enhance the performance of flexible electronics, providing a foundation for research, development, and manufacturing of more functionalities and applications.

### 4.1. Flexible Circuits and Reconfiguration Electronics

Due to the fluid and flexible properties of liquid metals, along with their outstanding electrical, thermal, and rheological characteristics, they demonstrate significant advantages in the field of printable flexible circuits and offer unique and innovative solutions [104]. Simultaneously, the preparation methods and techniques for patterning stretchable printed circuits based on liquid metals are crucial for expanding their application scope. The team led by K. Hjort at Uppsala University proposed a method involving laser etching of microchannels on silicone rubber, using polyvinyl alcohol as a peelable protective layer, followed by micron-scale liquid metal particle jet deposition [105]. This approach enables high-resolution liquid metal patterning while simultaneously achieving multi-layer connections and high-density component integration.

Methods for fabricating printed flexible circuits using liquid metals have been extensively explored and researched. Beyond mold casting [106,107], fundamental approaches for producing stretchable conductive flexible electronic devices include stencil printing [108], roller printing [109], transfer printing [110,111], direct-write printing [112], and inkjet printing [113] for depositing conductive patterns on flexible substrates. Additionally, flexible conductive fibers can be prepared via spinning [114] for diverse applications such as smart textiles and wearable sensors.

**Figure 4:**
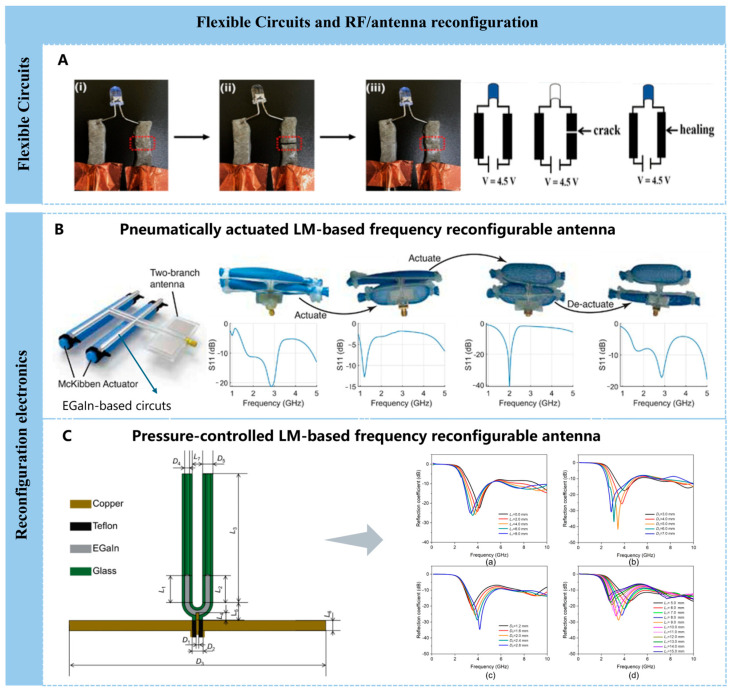
Applications in flexible circuits and reconfiguration electronics. (**A**) Photograph and schematic diagrams containing the PVA-LMPs-3 hydrogel with a connected LED indicator, (**i**) original hydrogel, (**ii**) furcate hydrogel, and (**iii**) self-healed hydrogel [99]. Copyright 2019, American Chemical Society. (**B**) Soft, dual-branch McKibben-actuated antenna enables broadband reconfiguration by independently tuning each arm, and the charts on the right show that each branch of the antenna can be driven to achieve different operating frequencies [117]. Copyright 2025, John Wiley and Sons. (**C**) Front cross-sectional view of the antenna and the influence of geometric parameters on its reflection coefficient, and the influence of geometric parameters on the antenna reflection coefficient: (a) L7, (b) D5, (c) D4 and (d) L [118]. Copyright 2022, MDPI.

Beyond the self-healing conductive connectivity inherent to liquid metal particles, printed flexible electronic devices also demand self-healing capabilities. Meihong Liao et al. found that the dynamic cross-links between the hydrogel and liquid metal particles can break and rejoin within the network, endowing the hydrogel with both electrical and mechanical self-healing capabilities (Figure 4A) [99]. As presented in Figure 4A, the PVA-LMPs-3 hydrogel was connected with the blue LED indicator. First, the LED indicator was lit by a power supply, as seen in Figure 4A(i). The LED light was completely switched off after the PVA-LMPs-3 hydrogel was severed, as shown in Figure 4A(ii). When bringing the two cleaved parts together, the hydrogel was healed automatically, and the LED indicator was lit again owing to the reformation of the dynamic cross-linking bonds at the contacted interfaces and the formation of the droplet-to-droplet connections of LMPs to reroute the electrically conductive path, as seen in Figure 4A(iii).

Driven by strategic demands for environmental sustainability and eco-friendly development, liquid metal flexible electronics must also be recyclable and reusable. Gallium and indium, as primary metallic elements in liquid metals, possess a high recycling value. An acid-leaching resin separation method for purifying gallium and indium [115] involves leaching liquid metal waste with nitric acid at 40 °C, followed by resin adsorption and elution, achieving recovery rates of 96.01% for gallium and 99.83% for indium.

The use of liquid metals enables the reconfigurable shape and the position of metallic components, thereby facilitating the fabrication of reconfigurable electronics for diverse applications. Typically, reconfigurable electronic devices are achieved by controlling the movement of liquid metals within microfluidic channels through electrochemical methods. The applied voltage potential determines the direction of movement, while the magnitude of current regulates the speed, ultimately forming controllable or even programmable functions [116]. To a certain extent, electrolytes in electrochemical processes can remove oxidation layers from liquid metals and alter their wettability. Simultaneously, surface modification of microfluidic channels significantly influences the generation, transport, separation, and coalescence of liquid metal droplets.

Benefiting from the inherent fluidity and electrical conductivity of liquid metals, liquid metal-based antennas can simultaneously achieve both flexibility and reconfigurability, thereby significantly broadening the functional adaptability and environmental resilience of the antennas. Yiwen Song et al. reported an omnidirectional liquid metal composite antenna system to enable high radiation efficiency when reconfiguring to different frequencies, with the aid of pneumatically actuated systems. The pneumatically actuated systems could result in a shape change that changes the electrical length of the antenna, which in turns influences its operating frequency (Figure 4B) [117]. Peng Qin et al. [118] reported a U-shaped dual-frequency-reconfigurable liquid metal monopole antenna. As shown in Figure 4C, the reconfiguration of the antenna’s operating frequency is achieved by dynamically adjusting the length of the liquid metal column within the U-tube structure via a precision syringe pump.

Beyond deformability and fluidity, liquid metals’ additional properties enable dynamic, multi-mode reconfigurable electronic devices. For instance, a nanophotonic platform combining a reconfigurable liquid metal substrate with a gold nanoantenna array achieves real-time tuning of high-resolution structural color patterns, applicable to dynamic optical displays, security anti-counterfeiting labels, and imaging applications [119].

### 4.2. Energy Storage and Energy Conversion

**Figure 5:**
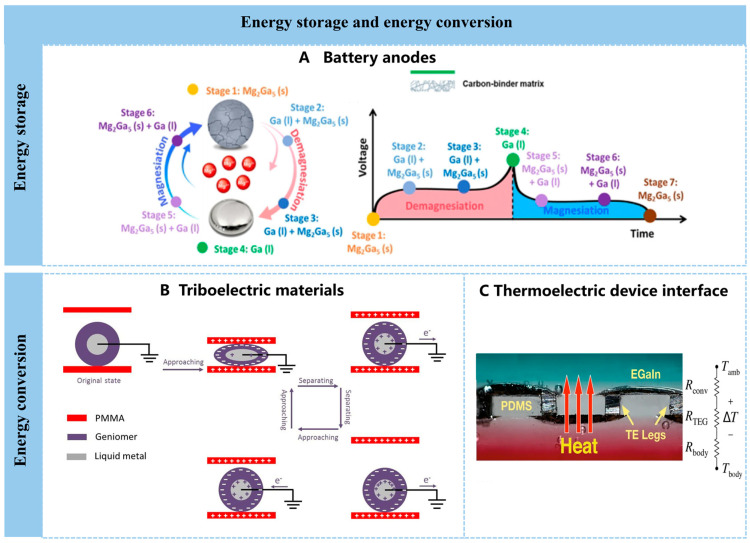
Applications in energy storage and energy conversion. (**A**) Schematic illustration of the states of charge and corresponding voltage profiles during the demagnesiation and remagnesiation of gallium-based electrode. Reprinted with permission from Wang, L. et al. [120] Copyright 2023, American Chemical Society. (**B**) Schematic illustration of the charge distribution of a TENG constructed from LM and PMMA [114]. Copyright 2020, Springer Nature. (**C**) Cross-sectional microscope image of a flexible TEG with liquid metal interconnections [126]. Copyright 2021, Springer Nature.

Traditional solid-state battery anodes suffer from structural or interfacial damage due to dendrite growth and volume changes, resulting in poor cycling performance. Self-healing electrodes made from liquid metals and alloys can overcome these issues through reversible liquid–solid phase transformations. The Eric Detsit team at the University of Pennsylvania employed liquid gallium as the active material in magnesium-ion battery anodes (Figure 5A) [120], which reversibly transforms into solid Mg_2_Ga_5_ during (de)magnesiumation cycles, achieving 1000 cycles. Liquid metals have also seen extensive research and application in lithium-ion batteries. Gallium-based lithium metal anode materials, leveraging their self-healing capability, fluidity, and metallic properties, can suppress destructive effects caused by significant volume expansion/contraction of anode and cathode materials. They also inhibit uncontrolled lithium dendrite growth to protect the lithium metal cathode [121]. Furthermore, the team of D.-H. Kim and H.-J. Koo at the Seoul National University of Science and Technology developed flexible perovskite solar cells using EGaIn electrodes. These cells consistently demonstrated stable performance during repeated bending cycles and the EGaIn electrodes were recoverable. Research also revealed that oxides on liquid metal surfaces significantly impact photovoltaic performance and impedance stability, acting as barriers to prevent metal diffusion while enhancing charge extraction and transfer through tunneling effects [122].

Stretchable supercapacitors offer excellent power performance, low heat generation, and ease of integration into clothing, making them a common energy storage solution for wearable electronics. Stretchable liquid metal supercapacitors can be fabricated by sandwiching a soft gel electrolyte layer between two liquid metal electrodes. A stretchable supercapacitor composed of liquid metal coated on polyester/neoprene textiles as current collectors, carbon nanotube layers as active materials, and gel electrolytes demonstrated a high areal capacitance of 527.8 mF·cm^−2^ and retained over 90% of its initial capacitance after 1000 repeated stretching cycles [123]. Overall, the integration of liquid metal electrodes not only enables efficient charge transfer but also confers significant structural stability, allowing these stretchable supercapacitors to conform to various shapes and undergo deformation without compromising performance.

Liquid metals can be used not only for fabricating energy storage devices but also for energy conversion systems. By converting mechanical energy into electrical energy and vice versa, they enable energy harvesting to power micro-sensors or wearable devices. Common principles for converting mechanical energy into electrical energy include the piezoelectric effect and the triboelectric effect. By incorporating liquid metal nanodroplets as nanofillers within a polyvinylidene fluoride matrix, the resulting liquid–solid/conductive-dielectric interface can significantly enhance both piezoelectric output and the reliability of such composites [124]. In addition, liquid metals have been developed as triboelectric materials, demonstrating significant performance potential in terms of conductivity, flexibility, high stability, and functionality [125]. A technique was developed to incorporate liquid metal nanodroplets into electrospun polyvinylidene fluoride-co-hexafluoropropylene (PVDF-HFP) nanofibers to enhance their triboelectric properties. Using a PVDF-HFP/2% LM nanofiber membrane as the negative triboelectric layer and thermoplastic polyurethane as the positive layer, the resulting triboelectric nanogenerator achieved peak open-circuit voltage and power density of 1680 V and 24 W m^−2^, respectively. This outstanding performance is attributed to multiple factors, including the introduction of LM nanodroplets, which enhance surface potential, capacitance, charge-trapping capability, and secondary polarization within the PVDF-HFP nanofibers. Chaoqun Dong et al. employed a thermal-drawing process to fabricate advanced elastomeric fibers that integrate micro-textured surfaces with multiple liquid metal electrodes, yielding microstructured stretchable triboelectric fibers whose efficiency rivals that of planar systems. This outstanding performance stems from several factors, including the introduction of deformable yet conductive liquid metal electrodes, enabling the fibers to maintain high electrical output even after repeated large deformations and to withstand strains up to 560%. The fibers can be woven into washable, deformable textiles delivering outputs of 490 V and 175 nC; beyond energy harvesting, they enable self-powered respiration monitoring and gesture sensing (Figure 5B) [114]

Padmanabhan Ramesh et al. recently reported a flexible thermoelectric generator (TEG) that provides efficient conversion of body heat to electrical energy (Figure 5C) [126]. The device relies on a low thermal conductivity aerogel–silicone composite that secures and thermally isolates the individual semiconductor elements that are connected in series using stretchable eutectic gallium–indium (EGaIn) liquid metal interconnections. The composite consists of aerogel particulates mixed into polydimethylsiloxane (PDMS), providing as much as a 50% reduction in the thermal conductivity of the silicone elastomer. Worn on the wrist, the flexible TEGs present output power density figures approaching 35 μWcm^−2^ at an air velocity of 1.2 ms^−1^, equivalent to walking speed. In this approach, the rigid legs are connected electrically in series using liquid metal interconnections embedded in a stretchable silicone elastomer.

### 4.3. Wearable Sensors

Due to the exceptional flexibility and electrical conductivity of liquid metals, they are also widely used in the fabrication of flexible sensors. For ease of understanding and summarization, liquid metal sensors are broadly categorized into mechanical sensors, skin surface electrophysiological signal sensors, and electrochemical biosensors.

**Figure 6:**
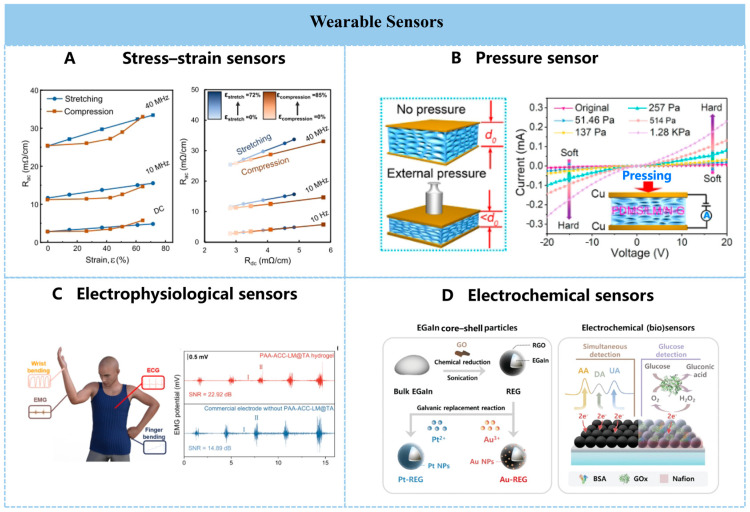
(**A**) Graph showing the distribution of changes in AC resistance per centimeter (Rac) and DC resistance per centimeter (Rdc) of liquid metal conductors under stretching and compressing actions at varied frequencies. Reprinted with permission from Rahman, M.S. et al. [127] Copyright 2022, Springer Nature. (**B**) Schematic diagram of the spongy pressure sensor’s thickness changing before and after pressure and its I-V curve under different pressures. Reprinted with permission from Li, Y. et al. [129] Copyright 2022, American Chemical Society. (**C**) Schematic illustration of human health monitoring based on liquid metal-based hydrogel electrophysiological signal sensor and comparison of its EMG signal detection ability with commercial gel electrodes. Reprinted with permission from Wei, J. et al. [130] Copyright 2025, American Chemical Society. (**D**) Schematic diagram of the simultaneous electrochemical detection of AA (ascorbic acid), DA (dopamine), UA (uric acid), and glucose by the deformable EGaIn electrode (RIDE/M-RIDE) composed of reduced graphene oxide assemblies and metal (Pt or Au)-decorated REG (M-REG). Reprinted with permission from Lee, D. et al. [133] Copyright 2023, John Wiley and Sons.

Mechanical sensors primarily encompass stress–strain sensors such as tensile and compressive types, which monitor sensing signals through changes in the sensor element’s resistance and capacitance. Conductive and mechanically stretchable materials based on liquid metal embedded in elastomer composites have been extensively studied for stretchable sensors. Liquid metal enhances low-power detection of mechanical stimuli in wearable electronics and quantitatively distinguishes mechanically deformed patterns traditionally indistinguishable, such as in-plane stretching and out-of-plane compression (Figure 6A) [127]. Simultaneously, liquid metals enhance the mechanical properties of elastomers. For instance, in a super-tough force sensor based on a liquid metal-polyvinyl alcohol composite, soft LM droplets significantly toughened the polyvinyl alcohol plastic, increasing toughness by 12.3 times while exhibiting remarkable mechanical durability [128].

Flexible and soft capacitive stress sensors are widely used in soft robotics, electronic skin, and human–machine interfaces due to their ideal temperature independence, low energy consumption, and excellent stability. Mixing LM particles into the elastomer increases its dielectric constant, effectively enhancing the sensitivity of capacitive sensors without hardening the elastomer composite. However, the limited compressible space within liquid metal elastomers severely restricts their range of mechanical stimulus detection as capacitive stress sensors. Therefore, designing and fabricating more advantageous three-dimensional structures is essential to improve sensor sensitivity. A liquid metal/nitrogen-doped graphene nanosheet hypersensitive pressure sensor sponge exhibits rapid response and recovery rates, with response/recovery times of 0.41/0.12 s and a comprehensive response range up to 476 kPa^−1^ (Figure 6B) [129].

Electrophysiological electrode patches are commonly used to collect surface electrophysiological signals for monitoring and evaluating human health. However, stable electrophysiological signal acquisition remains challenging due to electronic transmission losses and signal attenuation at the electrode–skin interface. A liquid metal-based hierarchical hydrogel, featuring outstanding adhesion, efficient self-healing capability, excellent stretchability, and conductivity, creates a conformal electrode/skin interface. This enables stable electrophysiological signal collection during motion (Figure 6C) [130]. Furthermore, the continuous improvement in the sensitivity of electrophysiological electrodes is inseparable from the rapid electron transport enabled by liquid metals and the flexibility that ensures conformal contact. A liquid metal with a weight fraction as high as 92% is stably anchored within a 3D continuous polymer network via electrostatic interactions in a dual-continuous thin-film electrode, achieving a sensitivity of 20 μV N^−1^ [131]. Electrophysiological signal sensors find broad applications in daily scenarios such as personal health monitoring, rehabilitation training assessment, and wearable human–machine interaction. Electrodes with high sensitivity can be further developed for monitoring sudden medical conditions (e.g., myocardial infarction abnormalities) or to better serve individuals with tactile impairments.

Electrochemical biosensors primarily detect chemical substances or biomolecules to obtain physiological parameters for health management. However, detection environments are often complex with numerous interfering factors, making it crucial to enhance detection specificity and sensitivity. A non-enzymatic glucose composite metal sensor utilizing in situ platinum plating on liquid metal enables specific recognition of glucose in sweat [132], opening possibilities for affordable, wearable personalized sweat monitoring. Surface modification of liquid metal can yield electrochemical biosensors capable of detecting diverse substances through more versatile detection methods. EGaIn core–shell particles assembled with reduced graphene oxide undergo further modification with metal nanoparticles via additional electrodeposition reactions. Leveraging their surface tunability, these particles simultaneously detect ascorbic acid, dopamine, and uric acid, alongside enzymatic glucose detection, demonstrating outstanding electrochemical sensing performance (Figure 6D) [133].

### 4.4. Microrobots

By combining liquid metals with other functional materials, flexible microrobots—also known as soft actuators—can be fabricated to respond to external fields. These actuators find applications across numerous fields. Selecting appropriate functional materials and designing high-performance composite systems involves choosing suitable drive mechanisms based on the target application. Subsequently, the appropriate device geometry is designed, encompassing device dimensions, equipment size and specifications, external shape (fibers, films, biomimetic structures, functional patterns, etc.), composite structures, and functional circuitry. Material preparation and processing are equally critical, encompassing composite mixing and fabrication, appropriate preparation techniques with controlled parameters, and assembly of composite devices. Finally, performance testing under corresponding drive modes is conducted on the completed devices to determine achievable material properties, strain rates, and lifetimes. For in vivo applications, heightened consideration must be given to the biocompatibility and stability of the material system to prevent irreversible damage or harm to the human body.

**Figure 7:**
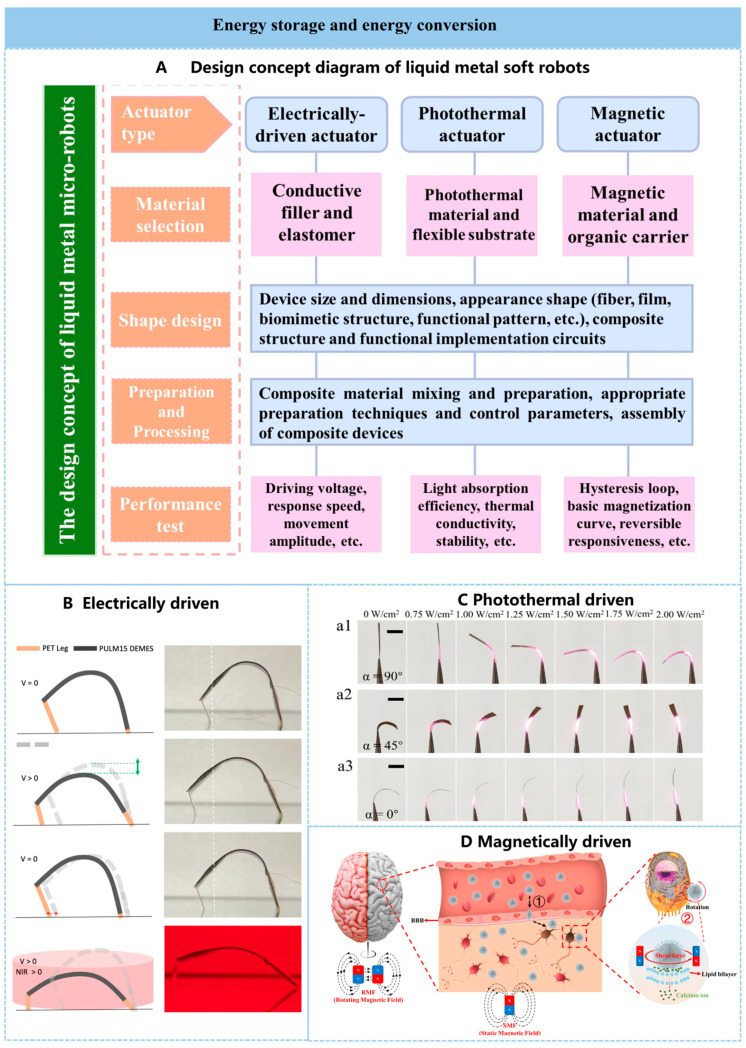
Manipulable microrobots. (**A**) Design concept diagram of liquid metal soft robots. (**B**) Schematic diagram and digital image of the working principle of the dual-anchor fixed dielectric minimum energy structure robot’s crawling mode controlled by voltage drive and near-infrared light assistance. Reprinted with permission from Tan, M.W.M. et al. [136]. Copyright 2022, Springer Nature. (**C**) Morphologies of the LM/PI/PTFE-5% photothermal actuator assembled with assembly angles of (**a1**) 90°, (**a2**) 45°, and (**a3**) 0° at different NIR laser intensities. Reprinted with permission from Li, X. et al. [138]. Copyright 2023, Wiley-VCH. (**D**) Schematic diagram of the deformation of magnetic liquid metal microrobots as they penetrate the blood–brain barrier under the influence of a magnetic field. Step 1 involves the shape-adaptive passage through the blood-brain barrier under the influence of the driving magnetic field; Step 2 involves applying magnetic mechanical stimulation to the cell membrane, which leads to the influx of calcium ions. Reprinted with permission from Wu, X. et al. [139].

The most common actuation method is electrically driven. Techniques for manipulating liquid metal interfacial tension using voltage include electrocapillarity, continuous electro-wetting, dielectric electro-wetting, and electrochemistry. These methods reduce the interfacial tension between liquid metal and surrounding electrolytes by driving charged particles (or, in electrochemical cases, chemical species) toward the interface. Such techniques enable manipulation and actuation of liquid metals at submillimeter length scales where interfacial forces dominate [134]. A liquid metal soft actuator featuring low drive voltage (0.5 V), low hysteresis, and operability across a wide pH range (0–14) in diverse solutions mimics muscle contraction and extension by leveraging the electrochemically tunable interfacial tension of liquid metal. Named the liquid metal artificial muscle, this actuator drives the tail fin of an unconstrained bionic robotic fish [135]. Beyond electrically driven actuation, multi-stimulus responsiveness can synergistically enhance actuator performance. A dielectroelastomer actuator exhibiting synergistic response to electric fields and near-infrared light incorporates liquid metal nanoparticles into carboxylated polyurethane elastomer. During co-stimulation, photothermal effects modulate the elastomer modulus to reduce driving electric fields while increasing energy density, achieving high toughness (55 MJ m^−3^) and superior driving performance (Figure 7B) [136]. An electro-thermal-near-infrared-magnetic triple-response actuator composed of cellulose nanofiber/polyvinyl alcohol/liquid metal (CNF/PVA/LM) and magnetic polydimethylsiloxane (MPDMS) layers exhibits thermoplastic transfer behavior, enabling controllable deformation fixation and recovery. This actuation approach enhances the actuator’s functionality and broadens its application scope [137].

The photothermal properties and flexibility of liquid metal microspheres enable the fabrication of photothermal actuators with enhanced responsiveness and diversified functionality. A liquid metal/polyimide/PTFE (LM/PI/PTFE) programmable photothermal actuator, along with the pronounced performance differences between PI and PTFE, endows the photothermal actuator with an outstanding response angle (130.74 ± 6.45°), response speed (46.62 ± 2.33°s^−1^), stability (2000 cycles over 10 h), and load-bearing capacity (Figure 7C) [138].

Magnetically driven liquid metal actuators represent a common and mature technology. These actuators are fabricated by combining liquid metal with magnetic materials to generate an externally controllable magnetic field for precise actuation and control. They not only deliver strong driving forces but also exhibit shape-changing capabilities in response to external environmental demands. One type of magnetic liquid metal encapsulates iron nanoparticles within LM droplets smaller than 10 μm, demonstrating high structural stability and robust magnetic mobility. It retains adaptive shape recovery capability even after 50% deformation, enabling vertical wall climbing up to 400% of its body length and passage through channels two-thirds its size. In vitro and in vivo experiments demonstrate that these magnetic liquid metal microrobots can adaptively pass through the blood–brain barrier under the influence of the driving magnetic field and exert mechanical force on the cell membranes, thereby causing calcium ion influx and achieving effective magnetic mechanical stimulation of neurons (Figure 7D) [139]. However, existing low-conductivity ferromagnetic materials readily lose magnetization once the external magnetic field is removed. The induced magnetic field primarily stems from the magnetic alignment of dispersed ferromagnetic particles, which can be easily demagnetized through mechanical disorder and reversibly reconfigured by re-aligning particles under weak magnetic fields. Based on this principle, a reconfigurable ferromagnetic liquid metal composite with high conductivity and deformability was prepared by uniformly mixing neodymium–iron–boron (NdFeB) particles into a gallium-based LM matrix. This material can serve as a printable conductive ink for paper electronics [140]. These inks were further applied in magnetic switches, flexible erasable magnetic recording paper, and magnetically actuated self-sensing paper-based soft robots. As previously mentioned, multiple energy-driven modes can enable additional functional requirements and application expansions. A dual-energy-transfer liquid metal elastomer composite responsive to magnetic fields can induce shape deformation using low-frequency (<100 Hz) field components while simultaneously powering onboard electronics and generating excess heat via radiofrequency (20 kHz–300 GHz) energy transfer. This enables novel functionality, offering a new approach for multifunctional microrobotics [141].

### 4.5. Thermal/Infrared Applications

The high thermal conductivity and dynamic infrared reflectance/emissivity of liquid metals also warrant attention. In recent years, their composite material properties have been effectively leveraged in thermal management and infrared-related fields. As traditional conduction and forced air convection technologies provide insufficient cooling for complex electronic systems, low-melting-point liquid metals or their alloys enhance heat transfer as coolants, significantly reducing chip temperatures [142] and addressing thermal management challenges posed by substantial heat generation in chips. Liu Jing’s team proposed the concept of nano-liquid metal fluids and employed several theoretical models to characterize and predict their properties. Their high electrical conductivity and electromagnetic drivability make them ideal base fluids for potential superconducting solutions [143], serving as excellent coolants for enhanced heat transfer applications. Additionally, liquid metal composites have been employed as thermal interface materials [144,145], combining outstanding flexibility with excellent thermal conductivity to enable effective thermal management for high-performance electronics and sensors. Simultaneously, gallium-based liquid metals have been developed for transformative electronic devices that can adjust shape and stiffness for specific purposes. They effectively reflect solar energy and radiate thermal energy, enabling device-level self-cooling temperature control in transformative electronics [146].

Secondly, liquid metals can be used to fabricate bionic tunable infrared emissivity coatings resembling cephalopods. Composite materials made from liquid metals and elastic substrates form a contracted droplet state with a corrugated surface under compressive strain, while exhibiting an expanded sheet-like state with a relatively smooth surface under no strain [147,148]. The reversible transition between these two states enables dynamic variation in the composite’s infrared reflectance/emissivity, thereby dynamically manipulating its infrared appearance. Non-contact infrared emissivity adjustment can be achieved via laser sintering. The photothermal energy from a CO_2_ laser penetrates liquid metal particles into the conductive network. As the laser energy density increases from 1.4 to 1.9 J cm^−2^, it causes the infrared emissivity to decrease from 0.4 to 0.24 in the 7.5–13 μm wavelength range. This reduction in emissivity stems from the loss of surface plasmon resonance effects in the infrared range following sintering of the liquid metal particles [149]. A liquid metal/silicone elastomer (Ecoflex) composite achieved changes in total reflectance and specular reflectance of approximately 44.8% and 61.2%, respectively [150]. Integrating this liquid metal composite with evaporated metal films enhances the system’s performance. This infrared-range color-changing capability finds applications not only in dynamic temperature regulation and flexible displays but also in infrared counter-detection technology, known as thermal camouflage. Due to liquid metal’s exceptional photothermal effect and low infrared emissivity, self-healing conductive liquid metal hydrogels hold significant potential for infrared camouflage [151]. Furthermore, photothermal infrared camouflage enables remote control and rapid response. Liquid metal/colorless polyimide films modified with polydopamine [152] not only exhibit outstanding photothermal effects enabling wide-range infrared camouflage from room temperature to 390 °C but also possess photoprogramming and photodriving capabilities due to glass transitions, residual stresses, and differing thermal expansion coefficients. This opens new strategies for dynamic infrared camouflage.

## 5. Development Trends and Future Outlook

The preceding section outlined the fundamental properties of liquid metals, strategies for preparing multifunctional composite materials, and their diverse applications. While existing research is highly diverse and innovative, a comprehensive review reveals several areas ripe for improvement. This paper identifies key challenges in current liquid metal research and offers perspectives on future development, aiming to provide guidance for subsequent studies.

As the preferred conductive material for flexible electronics, achieving a balance between conductivity and patterning poses a critical challenge in both research and application. Due to their high surface tension, patterning difficulties make it challenging to form intricate conductive patterns or circuits. Micro-/nano-structuring of liquid metals is a common solution, but oxidation increases with the degree of structuring, significantly reducing conductivity. This reduction contradicts the original intent of using liquid metals as flexible conductive materials, making the balance between conductivity and structuring critical.

Currently, most approaches to enhance conductivity involve adding a certain amount of other solid metal particles to liquid metal composites. However, the mixing method of these additional metallic elements with liquid metal, their impact on electron movement during conductivity, and the optimal addition quantity require clearer definitions and specifications. These added metal particles should serve as supplementary performance enhancers; otherwise, they risk replacing the inherent value of liquid metal itself.

In addition, research on functional material loading remains quite limited. Most researchers still focus on replacing different elastic matrices or incorporating common electrical materials like graphene and carbon nanotubes. Many unconventional functional materials should be incorporated into liquid metal multifunctional composites through innovative thinking and scientific design approaches, thereby expanding the functional capabilities and application boundaries of liquid metals. Research on liquid metals in catalysis has been constrained by their amorphous nature and limited observational methods. However, considering their catalytic properties could significantly leverage the inherent advantages of liquid metal materials.

Simultaneously, in this era of rapidly advancing intelligent algorithms, the development of liquid metal smart materials must keep pace with the times. Statistical analysis of existing research cases based on composite material properties and key performance metrics should be conducted. By accumulating extensive valid case data, standardized norms and reference criteria can be established, thereby creating a comprehensive liquid metal composite materials database. Integrating existing multidisciplinary theoretical computational models and intelligent algorithms will assist subsequent scholars in the design and experimental research of multifunctional liquid metal composites.

Finally, most current research and applications in flexible electronics remain confined to the laboratory stage. It is imperative to actively align with the standards of traditional solid-state electronics. While leveraging the inherent flexibility of liquid metals, corresponding key performance indicators must meet solid-state electronics benchmarks or reach equivalent baseline levels. Only when critical application metrics achieve existing industry standards will flexible electronics unlock broader market opportunities and developmental potential.

## Data Availability

No new data were created or analyzed in this study. Data sharing is not applicable to this article.

## References

[1] Agarwal R., Mohamad A. (2024). Gallium-based liquid metals as smart responsive materials: Morphological forms and stimuli characterization. Adv. Colloid Interface Sci..

[2] Wang X., He Y., Wu Y., Qi Z., Wang Y., Ding J., Zhang J., Fan Y., Wang H. (2024). The biocompatibility of gallium-based liquid metals with blood and serum. iScience.

[3] Khoshmanesh K., Tang S.Y., Zhu J.Y., Schaefer S., Mitchell A., Kalantar-Zadeh K., Dickey M.D. (2017). Liquid metal enabled microfluidics. Lab A Chip.

[4] Park J.E., Kang H.S., Baek J., Park T.H., Oh S., Lee H., Koo M., Park C. (2019). Rewritable, Printable Conducting Liquid Metal Hydrogel. ACS Nano.

[5] Ghasemian M.B., Tang J., Rahim M.A., Tang J., Kalantar-Zadeh K. (2024). Advances in liquid metal composites: Properties, applications, and future prospects. Trends Chem..

[6] Yu M., Cao C., Sa Z., Zhang C., Feng J., Sun Q., Ma X., Liang J., Sun Y., Yin R. (2025). Liquid metal alchemy: Unlocking self-healing gallium-based materials for next-generation electronics. Mater. Sci. Eng. R Rep..

[7] Gao W., Ota H., Kiriya D., Takei K., Javey A. (2019). Flexible Electronics toward Wearable Sensing. Acc. Chem. Res..

[8] Ju X., Velluvakandy R., Wu X., Merlos Rodrigo M.A., Heger Z., Bendíčková K., Frič J., Pumera M. (2025). Liquid Metal Microrobots for Magnetically Guided Transvascular Navigation. Adv. Mater..

[9] Chin R.M., Zhou L., Malakooti M.H. (2025). Solution-Processable, Elongated Liquid Metal Particles for Composites with Enhanced Thermal Management. ACS Appl. Mater. Interfaces.

[10] Daeneke T., Khoshmanesh K., Mahmood N., de Castro I.A., Esrafilzadeh D., Barrow S.J., Dickey M.D., Kalantar-zadeh K. (2018). Liquid metals: Fundamentals and applications in chemistry. Chem. Soc. Rev..

[11] Idrus-Saidi S.A., Tang J., Lambie S., Han J., Mayyas M., Ghasemian M.B., Allioux F.-M., Cai S., Koshy P., Mostaghimi P. (2022). Liquid metal synthesis solvents for metallic crystals. Science.

[12] Cao G., Liang J., Guo Z., Yang K., Wang G., Wang H., Wan X., Li Z., Bai Y., Zhang Y. (2023). Liquid metal for high-entropy alloy nanoparticles synthesis. Nature.

[13] Tang J., Zhao X., Li J., Guo R., Zhou Y., Liu J. (2017). Gallium-Based Liquid Metal Amalgams: Transitional-State Metallic Mixtures (TransM2ixes) with Enhanced and Tunable Electrical, Thermal, and Mechanical Properties. ACS Appl. Mater. Interfaces.

[14] Wang H., Chen S., Zhu X., Yuan B., Sun X., Zhang J., Yang X., Wei Y., Liu J. (2022). Phase transition science and engineering of gallium-based liquid metal. Matter.

[15] Zhu Y., Ding X., Jiang Y. (2024). Gallium-Based Liquid Metal Flexible Electronics Prepared by Solid–Liquid Phase Transition. J. Electron. Mater..

[16] Tang J., Zhang Z., Li L., Wang J., Liu J., Zhou Y. (2016). Influence of driving fluid properties on the performance of liquid-driving ejector. Int. J. Heat Mass Transf..

[17] He Y., You J., Dickey M.D., Wang X. (2023). Controllable Flow and Manipulation of Liquid Metals. Adv. Funct. Mater..

[18] Qi Y., Shen C., Hou Q., Ren Z., Jin T., Xie K. (2022). A self-healing liquid metal anode for lithium-ion batteries. J. Energy Chem..

[19] Jeong J., Seo J., Chung S.K., Lee J.-B., Kim D. (2020). Magnetic Field-Induced Recoverable Dynamic Morphological Change of Gallium-Based Liquid Metal. J. Microelectromech. Syst..

[20] Zhang Y., Xin Y., Zhao Q. (2025). Research and Application of Ga-Based Liquid Metals in Catalysis. Nanomaterials.

[21] Gu J., Zhang Y., Zhang Y., Shan L., Wu H., Xu H.-Y., Li D., He X., Dong L. (2025). Dynamic Interface Catalysis and Carbon Dioxide Reduction of Liquid Metal. Dalton Trans..

[22] Wu D., Liu D., Tian X., Lei C., Chen X., Zhang S., Chen F., Wu K., Fu Q. (2022). A Universal Mechanochemistry Allows On-Demand Synthesis of Stable and Processable Liquid Metal Composites. Small Methods.

[23] Guymon G.G., Malakooti M.H. (2022). Multifunctional liquid metal polymer composites. J. Polym. Sci..

[24] Ren L., Xu X., Du Y., Kalantar-Zadeh K., Dou S.X. (2020). Liquid metals and their hybrids as stimulus–responsive smart materials. Mater. Today.

[25] Hu Q., Hu F., Sun D., Zhang K. (2024). Responsive Gallium-Based Liquid Metal Droplets: Attributes, Fabrication, Response Behaviors, and Applications. Coatings.

[26] Chen S., Zhao R., Sun X., Wang H., Li L., Liu J. (2022). Toxicity and Biocompatibility of Liquid Metals. Adv. Healthc. Mater..

[27] Gancarz T. (2017). Density, surface tension and viscosity of Sn-Zn alloys with Ag, Bi, Ga and Na additions. Fluid Phase Equilibria.

[28] Shentu J., Pan J., Chen H., He C., Wang Y., Dodbiba G., Fujita T. (2023). Characteristics for Gallium-Based Liquid Alloys of Low Melting Temperature. Metals.

[29] Ruffman C., Lambie S., Steenbergen K.G., Gaston N. (2023). Structural and electronic changes in Ga–In and Ga–Sn alloys on melting. Phys. Chem. Chem. Phys..

[30] Kalantar-Zadeh K., Rahim M.A., Tang J. (2021). Low Melting Temperature Liquid Metals and Their Impacts on Physical Chemistry. Acc. Mater. Res..

[31] Wang L., Liu J. (2014). Liquid phase 3D printing for quickly manufacturing conductive metal objects with low melting point alloy ink. Sci. China Technol. Sci..

[32] Zhang X., Liu J., Deng Z. (2024). Bismuth-based liquid metals: Advances, applications, and prospects. Mater. Horiz..

[33] Krishnamurthi V., Vaillant P.H.A., Mata J., Nguyen C.K., Parker C.J., Zuraiqi K., Bryant G., Chiang K., Russo S.P., Christofferson A.J. (2024). Structural Evolution of Liquid Metals and Alloys. Adv. Mater..

[34] Blairs S. (2007). Review of data for velocity of sound in pure liquid metals and metalloids. Int. Mater. Rev..

[35] Handschuh-Wang S., Stadler F.J., Zhou X. (2021). Critical Review on the Physical Properties of Gallium-Based Liquid Metals and Selected Pathways for Their Alteration. J. Phys. Chem. C.

[36] Haynes W.M.E. (2011). CRC Handbook of Chemistry and Physics.

[37] Liu G.L. (2024). Thermal Properties of Liquid Metal.

[38] Mills K.C., Su Y.C. (2006). Review of surface tension data for metallic elements and alloys: Part 1–Pure metals. Metall. Rev..

[39] Zamora R., Martínez-Pastor J., Faura F. (2021). Thermal, Viscoelastic and Surface Properties of Oxidized Field’s Metal for Additive Microfabrication. Materials.

[40] Zuraiqi K., Zavabeti A., Allioux F.-M., Tang J., Nguyen C.K., Tafazolymotie P., Mayyas M., Ramarao A.V., Spencer M., Shah K. (2020). Liquid Metals in Catalysis for Energy Applications. Joule.

[41] Guan M., Huang Z., Bao Z., Ou Y., Zou S., Liu G. (2025). Gold nanoparticles incorporated liquid metal for wearable sensors and wound healing. Chem. Eng. J..

[42] Dickey M.D., Chiechi R.C., Larsen R.J., Weiss E.A., Weitz D.A., Whitesides G.M. (2008). Eutectic Gallium-Indium (EGaIn): A Liquid Metal Alloy for the Formation of Stable Structures in Microchannels at Room Temperature. Adv. Funct. Mater..

[43] Joshipura I.D., Ayers H.R., Majidi C., Dickey M.D. (2015). Methods to pattern liquid metals. J. Mater. Chem. C.

[44] Liu T., Sen P., Kim C.-J. (2012). Characterization of Nontoxic Liquid-Metal Alloy Galinstan for Applications in Microdevices. J. Microelectromech. Syst..

[45] Kramer R.K., Boley J.W., Stone H.A., Weaver J.C., Wood R.J. (2014). Effect of Microtextured Surface Topography on the Wetting Behavior of Eutectic Gallium–Indium Alloys. Langmuir.

[46] Hu L., Li J., Tang J., Liu J. (2017). Surface effects of liquid metal amoeba. Sci. Bull..

[47] Hirsch A., Michaud H.O., Gerratt A.P., de Mulatier S., Lacour S.P. (2016). Intrinsically Stretchable Biphasic (Solid–Liquid) Thin Metal Films. Adv. Mater..

[48] Guo R., Cui B., Zhao X., Duan M., Sun X., Zhao R., Sheng L., Liu J., Lu J. (2020). Cu–EGaIn enabled stretchable e-skin for interactive electronics and CT assistant localization. Mater. Horiz..

[49] Xing W., Wang H., Chen S., Tao P., Shang W., Fu B., Song C., Deng T. (2022). Gallium-Based Liquid Metal Composites with Enhanced Thermal and Electrical Performance Enabled by Structural Engineering of Filler. Adv. Eng. Mater..

[50] Yang J., Cao J., Han J., Xiong Y., Luo L., Dan X., Yang Y., Li L., Sun J., Sun Q. (2022). Stretchable multifunctional self-powered systems with Cu-EGaIn liquid metal electrodes. Nano Energy.

[51] Wang X., Zhao M., Zhang L., Li K., Wang D., Zhang L., Zhang A., Xu Y. (2022). Liquid metal bionic instant self-healing flexible electronics with full recyclability and high reliability. Chem. Eng. J..

[52] Seo Y., Kim H., Zan G., Song M.-J., Park C., Oh J.W., Kim Y.H., Choi S.-G., Shin E., Li S. (2025). Graft Copolymer-Stabilized Liquid Metal Nanoparticles for Lithium-Ion Battery Self-Healing Anodes. Adv. Funct. Mater..

[53] Hao Q., Tan X.F., Liu S., McDonald S.D., Yasuda H., Nogita K. (2023). In Situ Observation of the Ga- and Cu-Based Substrate Reaction by Synchrotron Microradiography. ACS Appl. Electron. Mater..

[54] Chen A., Wu B., Li L., You T., Wang J., Shen J., Pei C. (2023). Liquid metal embrittlement to boost reactivity and combustion performance of Al in composite propellants. Fuel.

[55] Jia M., Newberg J.T. (2019). Liquid–Gas Interfacial Chemistry of Gallium–Indium Eutectic in the Presence of Oxygen and Water Vapor. J. Phys. Chem. C.

[56] Jung W., Vong M.H., Kwon K., Kim J.U., Kwon S.J., Kim T.-i., Dickey M.D. (2024). Giant Decrease in Interfacial Energy of Liquid Metals by Native Oxides. Adv. Mater..

[57] Jeurgens L.P.H., Sloof W.G., Tichelaar F.D., Mittemeijer E.J. (2002). Structure and morphology of aluminium-oxide films formed by thermal oxidation of aluminium. Thin Solid Film..

[58] Xu Q., Oudalov N., Guo Q., Jaeger H.M., Brown E. (2012). Effect of oxidation on the mechanical properties of liquid gallium and eutectic gallium-indium. Phys. Fluids.

[59] Farrell Z.J., Tabor C. (2017). Control of Gallium Oxide Growth on Liquid Metal Eutectic Gallium/Indium Nanoparticles via Thiolation. Langmuir.

[60] Sutter E., Sutter P. (2012). Size-Dependent Room Temperature Oxidation of In Nanoparticles. J. Phys. Chem. C.

[61] Farrell Z.J., Jacob A.R., Truong V.K., Elbourne A., Kong W., Hsiao L., Dickey M.D., Tabor C. (2023). Compositional Design of Surface Oxides in Gallium–Indium Alloys. Chem. Mater..

[62] Bhuyan P., Singh M., Bae H., Kim T., Park S. (2025). Achieving exceptional elasto-dielectric properties in soft and stretchable elastomers through liquid metal particle incorporation: A comprehensive insight into fundamentals and multifaceted applications. Adv. Compos. Hybrid Mater..

[63] Wang D., Wang X., Rao W. (2021). Precise Regulation of Ga-Based Liquid Metal Oxidation. Acc. Mater. Res..

[64] Ding Y., Zeng M., Fu L. (2020). Surface Chemistry of Gallium-Based Liquid Metals. Matter.

[65] Zhang Y., Wang C., Tao J., Ma S., Xie S., Guo W., Bao R., Qiu J., Liu Y., Yang Z. (2025). Strain-Adaptive Liquid Metal Interfaces Overcome Poisson's Ratio Constraints in Piezoresistive Sensors for Infant Sleep Monitoring. Adv. Sci..

[66] Zhang W., Ou J.Z., Tang S.-Y., Sivan V., Yao D.D., Latham K., Khoshmanesh K., Mitchell A., O'Mullane A.P., Kalantar-zadeh K. (2014). Liquid Metal/Metal Oxide Frameworks. Adv. Funct. Mater..

[67] Lin Z., Qiu X., Cai Z., Li J., Zhao Y., Lin X., Zhang J., Hu X., Bai H. (2024). High internal phase emulsions gel ink for direct-ink-writing 3D printing of liquid metal. Nat. Commun..

[68] Tang L., Mou L., Zhang W., Jiang X. (2019). Large-Scale Fabrication of Highly Elastic Conductors on a Broad Range of Surfaces. ACS Appl. Mater. Interfaces.

[69] Liu S., Reed S.N., Higgins M.J., Titus M.S., Kramer-Bottiglio R. (2019). Oxide rupture-induced conductivity in liquid metal nanoparticles by laser and thermal sintering. Nanoscale.

[70] Liu S., Yuen M.C., White E.L., Boley J.W., Deng B., Cheng G.J., Kramer-Bottiglio R. (2018). Laser Sintering of Liquid Metal Nanoparticles for Scalable Manufacturing of Soft and Flexible Electronics. ACS Appl. Mater. Interfaces.

[71] Ozutemiz K.B., Wissman J., Ozdoganlar O.B., Majidi C. (2018). EGaIn–Metal Interfacing for Liquid Metal Circuitry and Microelectronics Integration. Adv. Mater. Interfaces.

[72] Kim J.-H., Kim S., Kim H., Wooh S., Cho J., Dickey M.D., So J.-H., Koo H.-J. (2022). Imbibition-induced selective wetting of liquid metal. Nat. Commun..

[73] Lee S., Jaseem S.A., Atar N., Wang M., Kim J.Y., Zare M., Kim S., Bartlett M.D., Jeong J.-W., Dickey M.D. (2025). Connecting the Dots: Sintering of Liquid Metal Particles for Soft and Stretchable Conductors. Chem. Rev..

[74] Xing R., Yang J., Zhang D., Gong W., Neumann T.V., Wang M., Huang R., Kong J., Qi W., Dickey M.D. (2023). Metallic gels for conductive 3D and 4D printing. Matter.

[75] Lee S., Lee G.-H., Kang I., Jeon W., Kim S., Ahn Y., Kim C.Y., Kwon D.A., Dickey M.D., Park S. (2025). Phase-change metal ink with pH-controlled chemical sintering for versatile and scalable fabrication of variable stiffness electronics. Sci. Adv..

[76] Zhou Z., Xing Z., Wang Q., Liu J. (2023). Electrochemical Oxidation to Fabricate Micro-Nano-Scale Surface Wrinkling of Liquid Metals. Small.

[77] Park G., Lee G.H., Lee W., Kang J., Park S., Park S. (2023). Divide and Conquer: Design of Gallium-Based Liquid Metal Particles for Soft and Stretchable Electronics. Adv. Funct. Mater..

[78] Tevis I.D., Newcomb L.B., Thuo M. (2014). Synthesis of Liquid Core–Shell Particles and Solid Patchy Multicomponent Particles by Shearing Liquids Into Complex Particles (SLICE). Langmuir.

[79] Boley J.W., White E.L., Kramer R.K. (2015). Mechanically Sintered Gallium–Indium Nanoparticles. Adv. Mater..

[80] Wu D., Wu S., Narongdej P., Duan S., Chen C., Yan Y., Liu Z., Hong W., Frenkel I., He X. (2024). Fast and Facile Liquid Metal Printing via Projection Lithography for Highly Stretchable Electronic Circuits. Adv. Mater..

[81] Lear T.R., Hyun S.-H., Boley J.W., White E.L., Thompson D.H., Kramer R.K. (2017). Liquid metal particle popping: Macroscale to nanoscale. Extrem. Mech. Lett..

[82] Zheng Y., Liu H., Yan L., Yang H., Dai L., Si C. (2024). Lignin-Based Encapsulation of Liquid Metal Particles for Flexible and High-Efficiently Recyclable Electronics. Adv. Funct. Mater..

[83] Nor-Azman N.-A., Ghasemian M.B., Fuchs R., Liu L., Widjajana M.S., Yu R., Chiu S.-H., Idrus-Saidi S.A., Flores N., Chi Y. (2024). Mechanism behind the Controlled Generation of Liquid Metal Nanoparticles by Mechanical Agitation. ACS Nano.

[84] Tutika R., Kmiec S., Haque A.B.M.T., Martin S.W., Bartlett M.D. (2019). Liquid Metal–Elastomer Soft Composites with Independently Controllable and Highly Tunable Droplet Size and Volume Loading. ACS Appl. Mater. Interfaces.

[85] Tang S.Y., Qiao R., Yan S., Yuan D., Zhao Q., Yun G., Davis T.P., Li W. (2018). Microfluidic Mass Production of Stabilized and Stealthy Liquid Metal Nanoparticles. Small.

[86] Hutter T., Bauer W.-A.C., Elliott S.R., Huck W.T.S. (2012). Formation of Spherical and Non-Spherical Eutectic Gallium-Indium Liquid-Metal Microdroplets in Microfluidic Channels at Room Temperature. Adv. Funct. Mater..

[87] Tian L., Ye Z., Gui L. (2021). A Study of Dielectrophoresis-Based Liquid Metal Droplet Control Microfluidic Device. Micromachines.

[88] Fang W.-Q., He Z.-Z., Liu J. (2014). Electro-hydrodynamic shooting phenomenon of liquid metal stream. Appl. Phys. Lett..

[89] Kang C., Roh S., Park H., Lee S., Lee T., Lee S.W., Kim H., Park J., Lee G., Park I. (2025). Needle-free, Liquid Metal-embedded Electrospray Deposition System for Controlled Microdroplet Printing. Adv. Sci..

[90] Chen Z., Lee J.B. (2019). Surface Modification with Gallium Coating as Nonwetting Surfaces for Gallium-Based Liquid Metal Droplet Manipulation. ACS Appl. Mater. Interfaces.

[91] Wang C., Gong Y., Cunning B.V., Lee S., Le Q., Joshi S.R., Buyukcakir O., Zhang H., Seong W.K., Huang M. (2021). A general approach to composites containing nonmetallic fillers and liquid gallium. Sci. Adv..

[92] Tian B., Wang Y., Jiang N., Chen Z., Zhang J. (2025). Chemical Exchange Between Silica Networks and Liquid Metals for All-Inorganic, Sintering-Free and Highly Conductive Inks. Adv. Mater..

[93] Li Y., Guo H., Xie Z., Fu Q. (2023). Effects of polymer polarity on the interface interaction of polymer/liquid metal composites. Chem. Commun..

[94] Xin Y., Peng H., Xu J., Zhang J. (2019). Ultrauniform Embedded Liquid Metal in Sulfur Polymers for Recyclable, Conductive, and Self-Healable Materials. Adv. Funct. Mater..

[95] Li X., Li M., Zong L., Wu X., You J., Du P., Li C. (2018). Liquid Metal Droplets Wrapped with Polysaccharide Microgel as Biocompatible Aqueous Ink for Flexible Conductive Devices. Adv. Funct. Mater..

[96] Huang Z., Zou S., Liu G. (2022). Surface Modification of Liquid Metal with p-Aniline Derivatives toward Bioapplications: Biosensing as an Example. ACS Appl. Mater. Interfaces.

[97] Huang Z., Guan M., Bao Z., Dong F., Cui X., Liu G. (2023). Ligand Mediation for Tunable and Oxide Suppressed Surface Gold-Decorated Liquid Metal Nanoparticles. Small.

[98] Zhang C., Allioux F.-M., Rahim M.A., Han J., Tang J., Ghasemian M.B., Tang S.-Y., Mayyas M., Daeneke T., Le-Clech P. (2020). Nucleation and Growth of Polyaniline Nanofibers onto Liquid Metal Nanoparticles. Chem. Mater..

[99] Liao M., Liao H., Ye J., Wan P., Zhang L. (2019). Polyvinyl Alcohol-Stabilized Liquid Metal Hydrogel for Wearable Transient Epidermal Sensors. ACS Appl. Mater. Interfaces.

[100] Wang D., Xu W., Liu Y., Chen L., He T., Tan H., He S., Zhu J., Wang C., Yu Z. (2025). Liquid Metal Gallium Pharmaceuticals. Theranostics.

[101] Qi Y., Miyahara M., Iwata S., Miyako E. (2024). Light-Activatable Liquid Metal Immunostimulants for Cancer Nanotheranostics. Adv. Funct. Mater..

[102] Lu Y., Hu Q., Lin Y., Pacardo D.B., Wang C., Sun W., Ligler F.S., Dickey M.D., Gu Z. (2015). Transformable liquid-metal nanomedicine. Nat. Commun..

[103] Shen Y., Xu X., Xing R., Wang Y., Su R., Kong J., Huang R., Dickey M.D., Qi W. (2025). pH-Switchable Multi-Enzyme-Mimicking via Liquid Metal Nanozyme. Small.

[104] Wu Y.-W., Alkaraki S., Tang S.-Y., Wang Y., Kelly J.R. (2023). Circuits and Antennas Incorporating Gallium-Based Liquid Metal. Proc. IEEE.

[105] Wang B., Prasad S., Hellman O., Li H., Fridberger A., Hjort K. (2023). Liquid Metal-Based High-Density Interconnect Technology for Stretchable Printed Circuits. Adv. Funct. Mater..

[106] Huang C., Wang X., Cao Q., Zhang D., Ding S., Xie H., Jiang J.-Z. (2022). Soft and Stretchable Liquid Metal–Elastomer Composite for Wearable Electronics. ACS Appl. Mater. Interfaces.

[107] Wu P., Wang Z., Yao X., Fu J., He Y. (2021). Recyclable conductive nanoclay for direct in situ printing flexible electronics. Mater. Horiz..

[108] Neumann T., Kara B., Sargolzaeiaval Y., Im S., Ma J., Yang J., Ozturk M., Dickey M. (2021). Aerosol Spray Deposition of Liquid Metal and Elastomer Coatings for Rapid Processing of Stretchable Electronics. Micromachines.

[109] Guo R., Yao S., Sun X., Liu J. (2019). Semi-liquid metal and adhesion-selection enabled rolling and transfer (SMART) printing: A general method towards fast fabrication of flexible electronics. Sci. China Mater..

[110] Guo R., Tang J., Dong S., Lin J., Wang H., Liu J., Rao W. (2018). One-Step Liquid Metal Transfer Printing: Toward Fabrication of Flexible Electronics on Wide Range of Substrates. Adv. Mater. Technol..

[111] Wang Q., Yu Y., Yang J., Liu J. (2015). Fast Fabrication of Flexible Functional Circuits Based on Liquid Metal Dual-Trans Printing. Adv. Mater..

[112] Zhu L., Zhou X., Zhang J., Xia Y., Wu M., Zhang Y., Lu Z., Li W., Liu L., Liu H. (2024). Self-Adhesive Elastic Conductive Ink with High Permeability and Low Diffusivity for Direct Printing of Universal Textile Electronics. ACS Nano.

[113] Karim N., Afroj S., Tan S., Novoselov K.S., Yeates S.G. (2019). All Inkjet-Printed Graphene-Silver Composite Ink on Textiles for Highly Conductive Wearable Electronics Applications. Sci. Rep..

[114] Dong C., Leber A., Das Gupta T., Chandran R., Volpi M., Qu Y., Nguyen-Dang T., Bartolomei N., Yan W., Sorin F. (2020). High-efficiency super-elastic liquid metal based triboelectric fibers and textiles. Nat. Commun..

[115] Li Z., Chen Z., Ma W., Cai C., Li S., Wang Y. (2023). Efficient separation and recovery of valuable gallium and indium from gallium-based liquid metal waste. J. Clean. Prod..

[116] Khan M.R., Trlica C., Dickey M.D. (2014). Recapillarity: Electrochemically Controlled Capillary Withdrawal of a Liquid Metal Alloy from Microchannels. Adv. Funct. Mater..

[117] Song Y., Bharambe A., Patel D.K., Zhuo B., Zadan M., Majidi C., Kumar S. (2025). Pneumatically-Actuated Liquid Metal-Based Frequency Reconfigurable Antenna. Adv. Sci..

[118] Qin P., Wang Q.-Y., Fu J.-H., Li C.-W., Zhang C.-L., Liu T.-Y., Gui L., Liu J., Deng Z.-S. (2022). A U-Shaped Dual-Frequency-Reconfigurable Monopole Antenna Based on Liquid Metal. Materials.

[119] Khan M.A.K., Vasini S., Divan R., Liu P.Q. (2025). Electrically Reconfigurable Liquid Metal Nanophotonic Platform for Color Display and Imaging. Adv. Mater..

[120] Wang L., Nelson Weker J., Family R., Liu J., Detsi E. (2023). Morphology Evolution in Self-Healing Liquid-Gallium-Based Mg-Ion Battery Anode. ACS Energy Lett..

[121] Zhang B.W., Ren L., Wang Y.X., Xu X., Du Y., Dou S.X. (2022). Gallium-based liquid metals for lithium-ion batteries. Interdiscip. Mater..

[122] Kim J.H., Kim D.H., Park N.G., Ko M.J., Cho J., Koo H.J. (2023). Liquid Metal-Based Perovskite Solar Cells: In Situ Formed Gallium Oxide Interlayer Improves Stability and Efficiency. Adv. Funct. Mater..

[123] Choi M.Y., Kim J.H., Kim S.K., Koo H.J., So J.H. (2023). Textile-Based Stretchable Supercapacitors with Liquid Metal Current Collectors. Adv. Funct. Mater..

[124] Liu J., Zeng S., Zhang M., Xiong J., Gu H., Wang Z., Hu Y., Zhang X., Du Y., Ren L. (2023). Giant Piezoelectric Output and Stability Enhancement in Piezopolymer Composites with Liquid Metal Nanofillers. Adv. Sci..

[125] Xu B., Peng W., He J., Zhang Y., Song X., Li J., Zhang Z., Luo Y., Meng X., Cai C. (2024). Liquid metal-based triboelectric nanogenerators for energy harvesting and emerging applications. Nano Energy.

[126] Padmanabhan Ramesh V., Sargolzaeiaval Y., Neumann T., Misra V., Vashaee D., Dickey M.D., Ozturk M.C. (2021). Flexible thermoelectric generator with liquid metal interconnects and low thermal conductivity silicone filler. Npj Flex. Electron..

[127] Rahman M.S., Huddy J.E., Hamlin A.B., Scheideler W.J. (2022). Broadband mechanoresponsive liquid metal sensors. npj Flex. Electron..

[128] Lou Y., Liu H., Zhang J. (2020). Liquid metals in plastics for super-toughness and high-performance force sensors. Chem. Eng. J..

[129] Li Y., Cui Y., Zhang M., Li X., Li R., Si W., Sun Q., Yu L., Huang C. (2022). Ultrasensitive Pressure Sensor Sponge Using Liquid Metal Modulated Nitrogen-Doped Graphene Nanosheets. Nano Lett..

[130] Wei J., Chen H., Pan F., Zhang H., Yang K., Yuan T., Fang Y., Ping H., Wang Q., Fu Z. (2025). Reusable Liquid Metal-Based Hierarchical Hydrogels with Multifunctional Sensing Capability for Electrophysiology Electrode Substitution. ACS Nano.

[131] Lu J., Li Q., Huang Q., Li D., Jiao Y., Wang Y., Li Y., Zou K., Chen Z., Gu J. (2024). A Highly Sensitive Surface Electrode for Electrophysiological Monitoring. Adv. Funct. Mater..

[132] Wang Y., Zheng J., Ma Y., Li M., Zhang S., Lu Y., Wang Q., Li Y. (2025). A novel flexible non-enzymatic composite-metal glucose detection sensor in sweat based on platinum in situ plating of liquid metal. Mater. Today Nano.

[133] Lee D., Park S., Seo J., Lee W.Y., Kim M.g., Kim J. (2023). Functionalized EGaIn Electrodes with Tunable Reduced-Graphene-Oxide Assembled EGaIn Core–Shell Particles for Soft and Deformable Electrochemical Biosensors. Adv. Funct. Mater..

[134] Eaker C.B., Dickey M.D. (2016). Liquid metal actuation by electrical control of interfacial tension. Appl. Phys. Rev..

[135] Shu J., Ge D.A., Wang E., Ren H., Cole T., Tang S.Y., Li X., Zhou X., Li R., Jin H. (2021). A Liquid Metal Artificial Muscle. Adv. Mater..

[136] Tan M.W.M., Bark H., Thangavel G., Gong X., Lee P.S. (2022). Photothermal modulated dielectric elastomer actuator for resilient soft robots. Nat. Commun..

[137] Li W., Sang M., Lou C., Liao G., Liu S., Wu J., Gong X., Ma Q., Xuan S. (2023). Triple-Responsive Soft Actuator with Plastically Retentive Deformation and Magnetically Programmable Recovery. ACS Nano.

[138] Li X., Du Y., Xiao C., Ding X., Pan X., Zheng K., Liu X., Chen L., Gong Y., Xue M. (2023). Tendril-Inspired Programmable Liquid Metal Photothermal Actuators for Soft Robots. Adv. Funct. Mater..

[139] Wu X., Zhang L., Tong Y., Ren L., Guo H., Miao Y., Xu X., Ji Y., Mou F., Cheng Y. (2024). Self-Adaptive Magnetic Liquid Metal Microrobots Capable of Crossing Biological Barriers and Wireless Neuromodulation. ACS Nano.

[140] Cao L., Yu D., Xia Z., Wan H., Liu C., Yin T., He Z. (2020). Ferromagnetic Liquid Metal Putty-Like Material with Transformed Shape and Reconfigurable Polarity. Adv. Mater..

[141] Zhang J., Soon R.H., Wei Z., Hu W., Sitti M. (2022). Liquid Metal-Elastomer Composites with Dual-Energy Transmission Mode for Multifunctional Miniature Untethered Magnetic Robots. Adv. Sci..

[142] Ma K., Liu J. (2007). Liquid metal cooling in thermal management of computer chips. Front. Energy Power Eng. China.

[143] Ma K.-Q., Liu J. (2007). Nano liquid-metal fluid as ultimate coolant. Phys. Lett. A.

[144] He Q., Qin M., Zhang H., Yue J., Peng L., Liu G., Feng Y., Feng W. (2024). Patterned liquid metal embedded in brush-shaped polymers for dynamic thermal management. Mater. Horiz..

[145] Chen H., Zhang H., Wang S., Yang B., Zhou K., Wu Y., Wang C., Tan S. (2025). Liquid metal enhanced thermal conduction of hydrogels for flexible cooling management. Chem. Eng. J..

[146] Byun S.H., Yun J.H., Heo S.Y., Shi C., Lee G.J., Agno K.C., Jang K.I., Xiao J., Song Y.M., Jeong J.W. (2022). Self-Cooling Gallium-Based Transformative Electronics with a Radiative Cooler for Reliable Stiffness Tuning in Outdoor Use. Adv. Sci..

[147] Yao B., Xu X., Han Z., Xu W., Yang G., Guo J., Li G., Wang Q., Wang H. (2023). Cephalopod-inspired polymer composites with mechanically tunable infrared properties. Sci. Bull..

[148] Yao B., He S., Wang R., Zeng Y., Shi W., Zhu Y., Xu X., Wang S., Wang Q., Wang H. (2025). Cephalopod-inspired soft composite with liquid metal inclusions for tunable infrared modulation. J. Mater..

[149] Im S., Frey E., Kim D.H., Heo S.Y., Song Y.M., Vong M.H., Jamalzadegan S., Wei Q., Gregg A.A., Khatib O. (2024). Tunable Infrared Emissivity Using Laser-Sintered Liquid Metal Nanoparticle Films. Adv. Funct. Mater..

[150] Zhang Y., Zhu H., An S., Xing W., Fu B., Tao P., Shang W., Wu J., Dickey M.D., Song C. (2024). Chameleon-inspired tunable multi-layered infrared-modulating system via stretchable liquid metal microdroplets in elastomer film. Nat. Commun..

[151] Li X., Jiang M., Du Y., Ding X., Xiao C., Wang Y., Yang Y., Zhuo Y., Zheng K., Liu X. (2023). Self-healing liquid metal hydrogel for human–computer interaction and infrared camouflage. Mater. Horiz..

[152] Pan X., Li X., Xiao C., Ding X., Zheng K., Liu X., Xue M., Chen L., Gong Y., Tian X. (2025). Light programmable liquid metal /colorless polyimide (LM/CPI) transparent film for dynamic infrared camouflage. Compos. Part B Eng..

